# A Brain‐Wide Atlas of Astrocytic Oxytocin Receptors Reveals a Glial Basis for Nucleus Accumbens Modulation of Affiliative Behavior

**DOI:** 10.1002/advs.202518450

**Published:** 2026-06-04

**Authors:** Clémence Denis, Stefan Stojilkovic, Kai‐Yi Wang, Cristina Márquez, Annabel C. Kleinwächter, Angel Baudon, Yuval Podpecan, Aurélia Ces, Mélanie Kremer, Isabelle Arnoux, Nathalie Rouach, Jemima Helen, Sophie Trender, Andreas Wallkum, Selina Wunsch, Franziska Schommer, Moritz C. Wimmer, Tim Schubert, Felix Franke, Jabir Aliyu Muhammad, Eva M. Eisemann, Ingrid Camila Possa‐Paranhos, Cassandra Baumann, Pierre‐Alexis Derrien, Quirin Krabichler, Cosmo Garcia, Henning Fröhlich, Matthew K. Kirchner, Valery Grinevich, Pascal Darbon, Javier E. Stern, Ferdinand Althammer, Alexandre Charlet

**Affiliations:** ^1^ Centre National de La Recherche Scientifique and University of Strasbourg Institute of Cellular and Integrative Neuroscience Strasbourg France; ^2^ Institute of Human Genetics Heidelberg University Heidelberg Germany; ^3^ Center For Neuroscience and Cell Biology CNC‐UC University of Coimbra Coimbra Portugal; ^4^ Centre For Innovative Biomedicine and Biotechnology University of Coimbra Coimbra Portugal; ^5^ Center for Interdisciplinary Research in Biology Collège de France, CNRS, INSERM, PSL‐Neuro, Université PSL Paris France; ^6^ Department of Neuropeptide Research in Psychiatry Central Institute of Mental Health German Center for Psychiatry Medical Faculty Mannheim University of Heidelberg Mannheim Germany; ^7^ Center For Neuroinflammation and Cardiometabolic Diseases, and Neuroscience Institute Georgia State University Atlanta Georgia USA; ^8^ International Joint Laboratory for Translational Research on Neuromodulation, Shenzhen Institutes of Advanced Technology Chinese Academy of Sciences Shenzhen China

**Keywords:** astrocyte, atlas, behavior, nucleus accumbens, oxytocin

## Abstract

Until recently, it was widely assumed that oxytocin signaling occurred exclusively through the activation of neuronal oxytocin receptors, with neurons being the primary targets of released oxytocin. However, this view was challenged by the discovery of functional oxytocin receptors in central amygdala astrocytes, which are essential for the proper function of local neuronal microcircuits. Since then, astrocytic oxytocin receptors have been implicated in various aspects of rodent physiology and behavior, yet it remains unclear whether this mechanism is region‐specific or widespread across the brain. Here, we provide extensive anatomical data on oxytocin receptor expression in mice and rats, functionally validated through calcium imaging. Based on this mapping and using genetic, calcium imaging and behavioral approaches, we further demonstrate a critical role for oxytocin receptor‐expressing astrocytes in the nucleus accumbens in social behavior. In summary, our findings demonstrate that oxytocin receptors are widely expressed in astrocytes across different brain regions. In the nucleus accumbens, these receptors modulate social behavior—an observation with significant implications for the current model of oxytocinergic modulation in the brain.

## Graphical abstract

The cellular actors of oxytocin signaling are under intense scrutiny. A brain‐wide anatomical and functional analysis in mice and rats reveals widespread expression of oxytocin receptors in astrocytes. These receptors are functionally active and, in the nucleus accumbens, selectively regulate male social affiliation. The findings confirm astrocytes as key cellular actors of oxytocin signalling in the mammalian brain.

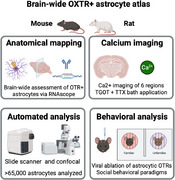

## 1 Introduction

Oxytocin (OT), a neuropeptide traditionally associated with social bonding, reproduction, and stress regulation, plays a pivotal role in modulating neural circuits and behaviors across species [1, 2]. Beyond its well‐documented functions, OT exemplifies the broader significance of neuropeptide systems in regulating brain functions and behaviors [3]. Understanding the OT system not only deepens our knowledge of social and emotional behaviors but also provides a framework for investigating how neuropeptides shape brain networks beyond classical neurotransmitter systems. For example, OT is released both in a paracrine fashion through volume transmission and somatodendritic release [4, 5] in addition to targeted axonal release at synapses [6], underscoring the unique functional identity of OT neurons. OT receptors (OTRs) are widely distributed in the CNS [4] and are key players in fine‐tuning neuronal and glial interactions, the latter being an area of research that remains relatively unexplored.

While its effects have been extensively studied in neurons, emerging evidence suggests that OT also influences non‐neuronal cells, particularly astrocytes, which are increasingly recognized as active contributors to brain function and plasticity [7]. Astrocytes, once considered mere supportive cells, are now known to regulate synaptic transmission, neurovascular coupling, and network homeostasis, raising intriguing questions about their role in OT‐mediated processes [8, 9]. Additionally, astrocytic networks communicate through calcium signaling, impacting neuronal oscillations and circuit dynamics [10], which may be particularly relevant for neuropeptide systems like OT that modulate social and affective behaviors.

Although earlier studies suggested that OTRs may be present in non‐neuronal cells [11], more recent work has provided direct anatomical and functional evidence that astrocytes express functional OTRs. A landmark study demonstrated the presence of functional OTR on astrocytes in the amygdala [12]. Here, OTR‐expressing astrocytes (OTR+ astrocytes) modulate synaptic activity and behavioral outcomes to regulate emotional valence and anxiety [12] and are responsible for neuroglial morphofunctional plasticity in response to acute stress [13]. Furthermore, the growing evidence for astrocytic involvement in OT signaling emphasizes the need to explore the broader implications of these interactions across different brain regions [14]. Interestingly, compelling data suggested that OT astrocyte interactions extend beyond the amygdala [15, 16]; [17, 18], reinforcing the notion that astrocytes play an integral role in mediating OT ’s diverse physiological effects.

However, despite these advances, the distribution and functional roles of OTR+ astrocytes across different brain regions remain largely unexplored, leaving a significant gap in our understanding of the broader implications of OT‐astrocyte interactions. Addressing this gap requires a systematic mapping of the anatomical and functional landscape of OTR+ astrocytes throughout the brain. The present study aims to characterize the distribution and functional properties of OTR‐expressing astrocytes in the rodent (mice and rats) brain, laying the groundwork for future investigations into their contributions to OT‐dependent processes.

In this study, using a combinatory approach—including RNAscope, immunohistochemistry, Ca^2^^+^ imaging, virus‐mediated astrocyte‐specific OTR ablation, and behavioral tests—we demonstrate the widespread distribution across the surveyed regions of OTR+ astrocytes and their physiological and behavioral significance. Our findings reveal that OTR+ astrocytes are present across all the OTR‐expressing regions surveyed.

In particular, we focused on deciphering the role of nucleus accumbens (NAc) astrocytes. While the role of neuronal OTRs in the NAc is documented [19, 20, 21], the involvement of astrocytic OTR signaling in this region remains less clear. Astrocytes in the NAc respond to neurotransmitters like dopamine with intracellular calcium transients and gliotransmitter release, thereby modulating synaptic transmission [22]. Given the well‐known involvement of the NAc in social behaviors, we speculated that astrocytic OTRs may be involved. Here, we found that NAc astrocytic OTRs contribute to male‐specific affiliative social interaction, particularly nose‐to‐nose investigation, and to social memory. Thus, our findings raise questions about the prevailing dogma regarding the necessity and extent of neuronal OTR activation and offer insights into OTR‐based modulation of physiology and behavior, highlighting possible OT‐based targets for future therapeutic investigation.

## 2 Results

### 2.1 Validation of the Experimental Pipeline and Detection of Astrocytic OTRs

**FIGURE 1:**
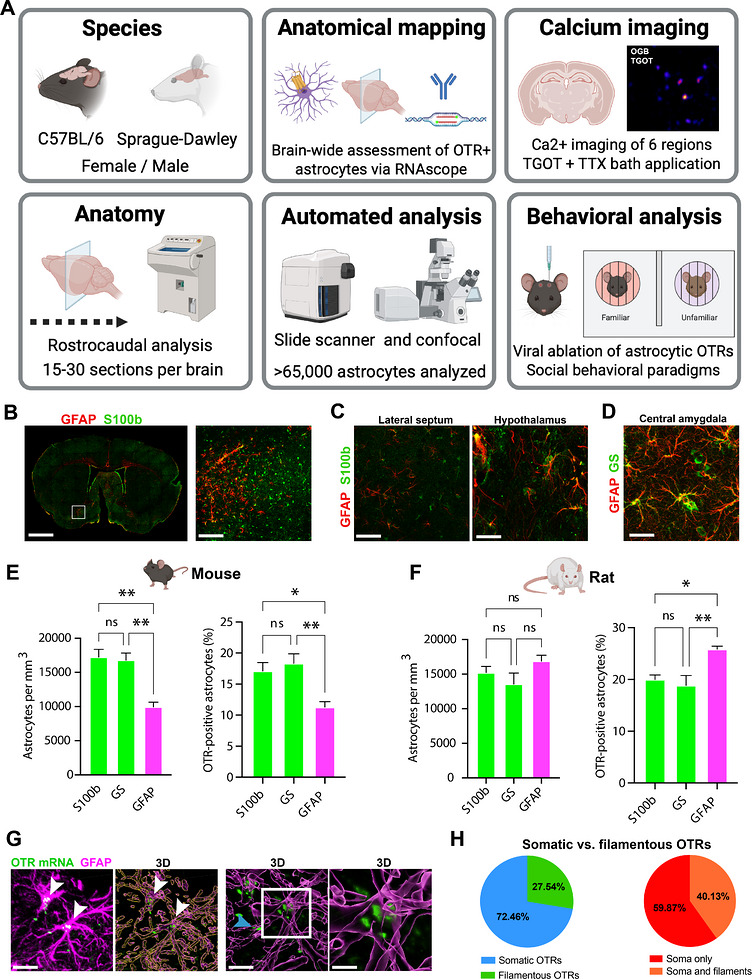
Schematic overview and experimental validation of the OTR+ astrocyte atlas. (A). Schematic illustration of the individual components and approaches used to generate the astrocyte OTR atlas. (B). Overview image highlighting different distribution of glial markers GFAP and S100b in the mouse brain. Scale bar = 2 mm. (C). Representative images of mouse LS and hypothalamus stained with GFAP and S100b. Scale bars = 200 and 50 µm. (D). Representative confocal image showing rat NAc labeled with astrocyte markers GFAP and GS. Scale bar = 50 µm. (E). Quantification of astrocyte numbers and OTR+ astrocytes using different glial markers in mice (LS, NAc, and CeA pooled, *n* = 4 images per region, *n* = 6 animals, 3male/female). One‐way ANOVA followed by Tukey test. (F) Quantification of astrocyte numbers and OTR+ astrocytes using different glial markers in rats (LS, NAc, and CeA pooled, *n* = 4 images per region, *n* = 6 animals, 3male/female). One‐way ANOVA followed by Tukey test. (G) RNAscope visualization of OTR reveals mRNA transcripts in astrocyte somata and filaments. Images show a representative example of a rat CeA astrocyte depicted as raw fluorescent confocal image and three‐dimensional reconstruction. White arrowheads indicate OTR mRNA in cell bodies, blue arrowheads in astrocyte filaments. (H) Pie charts show percentages of somatic and filamentous OTRs (left) and relative percentages of astrocytes displaying only somatic or somatic and filamentous OTRs. The left pie chart represents a subset of the data shown in the right chart (40.3%). Scale bars = 50, 25, and 5 µm. LS—Lateral Septum, NAc—nucleus accumbens, CeA—central amygdala. Data are expressed as mean across animals ± SEM. ^*^ *p* < 0.05, ^**^ *p* < 0.01, ^***^ *p* < 0.001. Detailed statistics can be found in Statistic Table S1.

In this project we aimed to perform a comprehensive anatomical and functional analysis of astrocytic OTRs in mice and rats combined with functional Ca^2+^ imaging (Figure 1A). To this end, we first assessed the validity and reliability of an automated RNAscope pipeline (Figure 1B–F). We first performed a baseline assessment using the astrocyte‐specific markers glutamine synthetase (GS), S100b and glial fibrillary acidic protein (GFAP) (Figure 1B–D and Figure S1A,B). We analyzed their presence in various brain regions such as the lateral septum (LS), hypothalamus, central amygdala (CeA) and NAc of mouse and rat brains. While GS and S100b antibodies yielded comparable results in mouse brain tissue, we observed significantly fewer GFAP‐positive cells (Figure 1E). In rats, we did not detect differences in the number of GFAP, GS or S100b astrocytes (Figure 1F).

Next, for all antibodies and mRNA probe combinations, we further assessed reliability (compared to manual human analysis) and inter‐animal variation in both species (Figure S1). In mice, individual combination of these markers with RNAscope against OTR mRNA resulted in significantly fewer putative GFAP OTR+ astrocytes than GS or S100b OTR+ astrocytes (Figure 1E). On the other hand, in rats, we observed significantly more OTR+ astrocytes using the GFAP antibody compared to sections stained with GS and S100b antibodies (Figure 1F). Finally, in rats, we further compared GFAP protein with GFAP mRNA and found significantly fewer OTR+ astrocytes using mRNA (Figure S1A, B).

**FIGURE 2:**
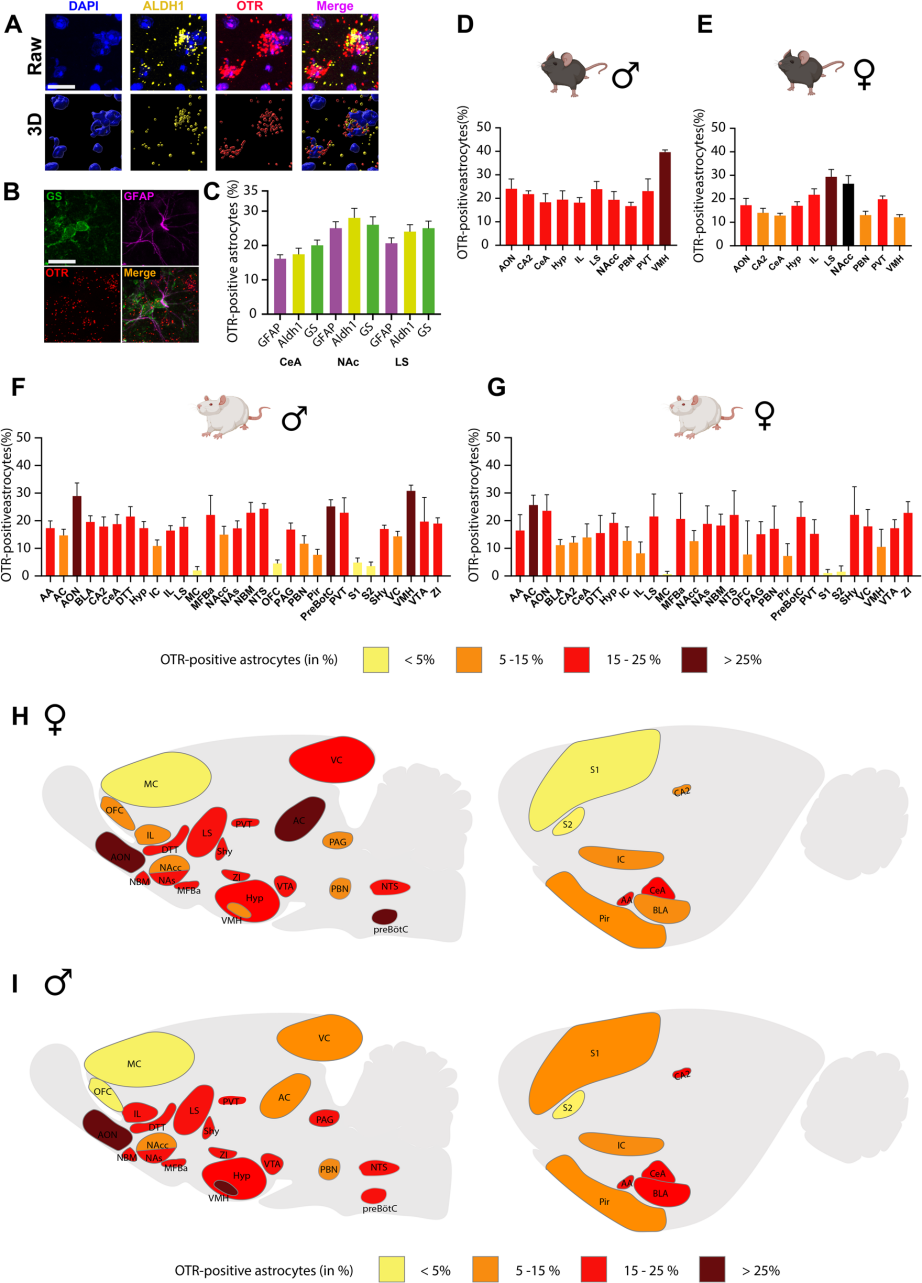
Widespread mapping of OTR+ astrocytes in rats and mice of both sexes. (A). Panels show raw and three‐dimensionally reconstructed OTR+ astrocytes in mice identified via multiplexed RNAscope against Aldh1L1 and OTR mRNA. Scale bar = 25 µm. (B). Confocal images highlight co‐localization of GS, GFAP and OTR mRNA in mice. Scale bar = 25 µm. (C) Validation of GFAP, Aldh1L1 and GS approaches to detect and quantify OTR+ astrocytes across CeA, LS, and NAc, *n* = 4 mice per group (D). Quantification of OTR+ astrocytes in male mice (*n* = 4 per group). (E). Quantification of OTR+ astrocytes in female mice (*n* = 4 per group). (F). Quantification of OTR+ astrocytes in male Sprague–Dawley rats (*n* = 4 per group). (G). Quantification of OTR+ astrocytes in female Sprague‐Dawley rats (*n* = 4 per group). (H,I). Schematic illustration of OTR+ astrocyte distribution in the male and female rat. Different colors represent varying expression levels across different brain regions analyzed. AA‐Anterior Amygdala, AC‐Auditory Cortex, AON‐Anterior Olfactory Nucleus, BLA‐Basolateral Amygdala, CA2‐dorsal Cornu Ammonis of Hippocampus, CeA‐Central Amygdala, DTT‐Dorsal tenia tecta, Hyp‐Hypothalamus, IC‐Insular Cortex, IL‐Infralimbic cortex, LS‐Lateral Septum, MC‐Motor Cortex, MFBa‐Medial Forebrain Bundle a, NAcc‐Nucleus Accumbens core, NAs‐Nucleus Accumbens shell, NBM ‐ Nucleus basalis of Meynert, NTS‐Nucleus Tractus Solitaris,  OFC‐Olfactory Cortex, PAG‐Periaqueductal grey, PBN‐Parabrachial Nucleus, Pir‐Piriform cortex, preBötC‐preBötzinger Complex, PVT‐Paraventricular thalamic nucleus, S1– Somatosensory Cortex 1, S2– Somatosensory Cortex 2, SHy‐Septohypothalamic nucleus, VC‐Visual Cortex, VMH‐Ventromedial Hypothalamus, VTA‐Ventral tegmental Area, ZI‐Zona inserta. Data are expressed as mean across animals ± SEM. Detailed statistics can be found in Statistic Table S2. ^****^ *p* < 0.0001.

Although the proportion of GFAP+OTR+ astrocytes differed between mice and rats, this difference did not affect the interpretation of the subsequent analyses. Our comparisons across regions and species focused primarily on the presence and distribution of OTR‐expressing astrocytes rather than on absolute percentages. Therefore, the observed species differences in marker proportions were interpreted cautiously and do not alter the overall conclusion that astrocytic OTRs are broadly distributed across the surveyed brain regions in both species.

Next, we wanted to probe whether OTR mRNA is present in both the soma and filaments. To this aim, we performed three‐dimensional reconstruction [25] of astrocytes in three selected regions (i.e. NAc, LS, and CeA) labeled via GFAP antibodies and processed via RNAscope against OTR mRNA (Figure 1G and Figure S1). Intriguingly, we found that 40.13% of OTR+ astrocytes contained OTR mRNA within their processes (Figure 1H). Within this OTR+ astrocyte subpopulation, OTR mRNA within processes accounted for 27.54% of total OTR mRNA. These findings suggest the presence of OTR mRNA and local OTR protein translation in astrocytic first‐order processes.

### 2.2 Widespread Distribution of OTR+ Astrocytes in OTR‐Expressing Brain Regions

To further validate our pipeline, given the debated specificity of GFAP as an astrocyte marker [21, 22], we examined three consistent brain regions across male and female mice and rats (CeA, LS, and NAc). We used multiplexed RNAscope with aldehyde dehydrogenase 1 family member L1 (Aldh1l1) and OTR mRNA, as well as immunohistochemical staining for GS or GFAP combined with OTR mRNA (Figure 2A–C). Both approaches showed no significant regional differences (Figure 2C), supporting the reliability of the GFAP labelling as an astrocyte marker for such large screening study.

Next, we systematically probed for the presence of OTR+ astrocytes in brain regions with known OTR expression along the rostro‐caudal gradient. To do so, we analyzed 31 brain regions in rats and 10 in mice (Figure 2 and Figure S2). The percentage of non‐astrocytic cells expressing OTR range 2.3% to 33.6% (Figure S2). Unexpectedly, on the other hand, we detected in both male and female rats OTR expressing astrocytes in every single brain region known to express the OTR, except for the somatosensory cortex (Figure 2D‐G). Indeed, the percentage of astrocytes expressing OTRs range from 0.7% to 39.6%, which were predominantly selected based on their relative levels of OTR abundance, but also include several cortical regions for a near‐complete map of the rodent brain (Figure 2). Interestingly, relative numbers of OTR+ astrocytes were consistent both across species and sexes (Figure 2D–G), with the exception of few regions as the anterior olfactory nucleus (AON), auditory cortex (AC), preBötzinger complex (preBötC) and ventromedial hypothalamus (VMH). Notably, in the VMH, in both species the numbers of OTR+ astrocytes were 3 times higher in males than in females (9.6% vs. 34.7% in rats, 13.2 vs 39.6% in mice; *p* < 0.0001, two‐tailed *t*‐test). This suggests a sex‐specific physiological function of these cells in these regions. All other regions investigated showed comparable levels between both sexes and no significant differences between species were observed for any of the investigated brain regions (Figure 2D–G). Of note, high numbers (>20%) of OTR+ astrocytes were detected in the hypothalamus, lateral septum (LS), infralimbic cortex, anterior olfactory nucleus and paraventricular nucleus of the thalamus in both mice and rats. These anatomical results for male and female rats are summarized in the schematic in Figure 2H,I.

In conclusion, our results suggest that OTR expressing astrocytes are present in all brain regions expressing OTR, yet in various proportions.

### 2.3 Anatomo‐Functional Analysis of OTR+ Astrocytes in the Rat Brain

**FIGURE 3:**
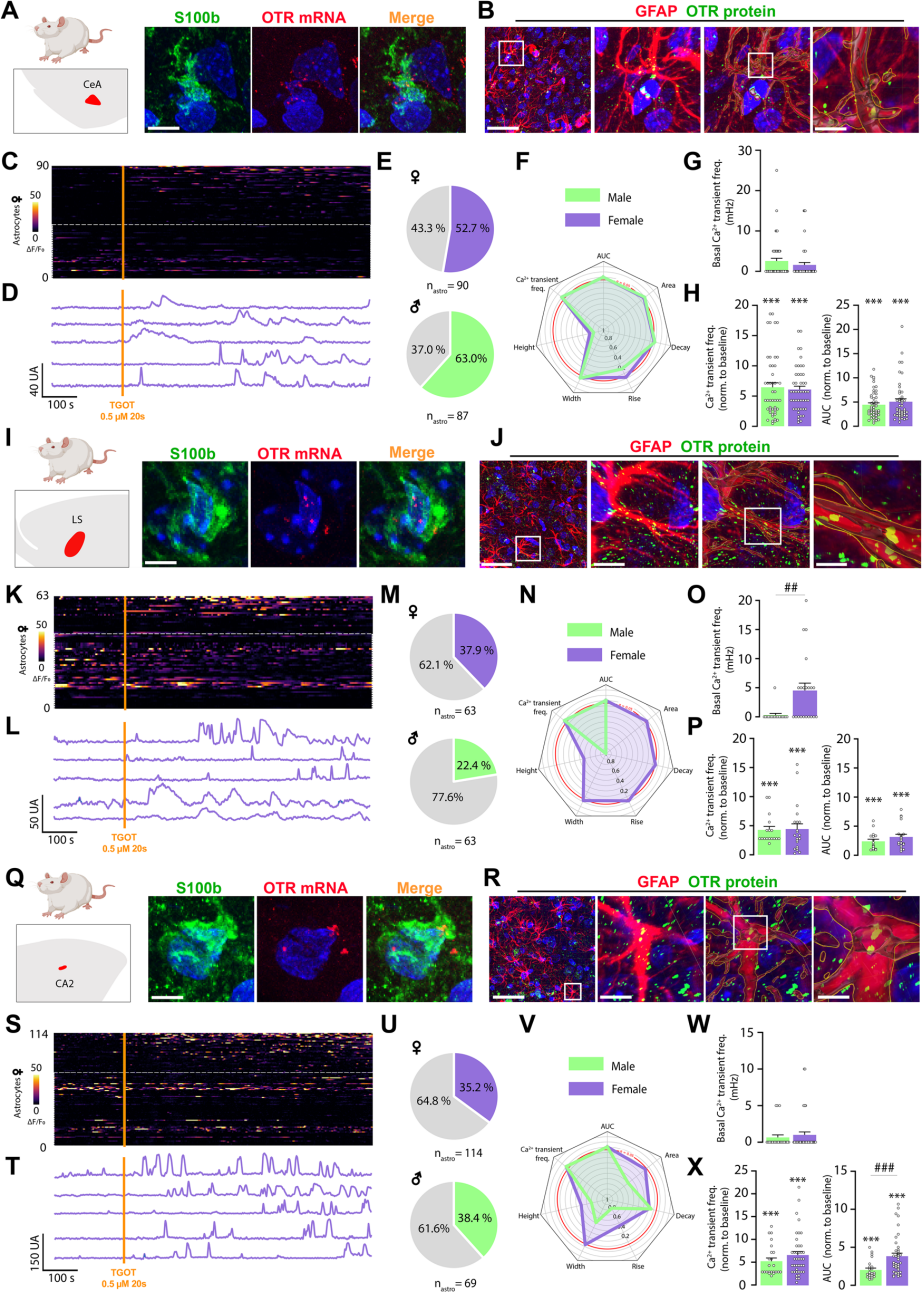
Anatomical mapping and functional assessment of OTR+ astrocytes in rat CeA, LS, and CA2. (A–H). OTR mRNA positive astrocytes in the rats CeA and their response to OTR activation. (A). High magnification confocal image shows overlap of S100b and OTR mRNA in the CeA of a female rat. Scale bar = 10 µm (B). Dual immunostaining against GFAP and OTR protein reveals the presence of OTRs in somata and filaments of rat astrocytes within the CeA. Three‐dimensional reconstruction (images 3 and 4) demonstrates expression of OTR within GFAP‐positive processes. Scale bars = 100, 10, and 1 µm. (C). Heatmap of OGB1 fluorescence intensity of individual astrocytes in response to TGOT (500 nm) in a female. The vertical line indicates 20s of TGOT application. (D). Typical ΔF traces of OGB1 fluorescence intensity of individual astrocytes in response to TGOT (500 nm) + TTX (1 µm) in females. The vertical line indicates 20s of TGOT application. (E). Proportion of CeA astrocytes responding to TGOT application in male and female individuals (n_astro male CeA_ = 87, n_record male CeA_ = 12, n_astro female CeA_ = 90, n_record female CeA_ = 31, n_rat CeA_ = 3–8). (F). Comparison of calcium activity triggered by TGOT application in male and female individuals. Spider plots showing the p‐value of the comparison between the basal state and after the application of TGOT for different calcium signaling parameters. (G). Bar plot showing mean frequency of calcium transients in male and females during baseline time (n_astro resp male CeA_ = 54, n_record resp male CeA_ = 12, n_astro resp female CeA_ = 47, n_record resp female CeA_ = 31, n_rat resp CeA_ = 3‐8). (H). Bar plots showing mean AUC and mean frequency of calcium transients triggered by TGOT application in males and females normalized to baseline values (n_astro resp male CeA_ = 54, n_record resp male CeA_ = 12, n_astro resp female CeA_ = 47, n_record resp female CeA_ = 31, n_rat resp CeA_ = 3–8). (I–P). OTR mRNA positive astrocytes in the rats LS and their response to OTR activation. (I). High magnification confocal image shows overlap of S100b and OTR mRNA in the LS of a female rat. Scale bar = 10 µm (J). Dual immunostaining against GFAP and OTR protein reveals presence of OTRs in somata and filaments of rat astrocytes within the LS. Three‐dimensional reconstruction (images 3 and 4) demonstrates expression of OTR within GFAP‐positive processes. Scale bars = 100, 10, and 1 µm. (K). Heatmap of OGB1 fluorescence intensity of individual astrocytes in response to TGOT (500 nm) in female rats. The vertical line indicates 20s of TGOT application. (L). Typical ΔF traces of OGB1 fluorescence intensity of individual astrocytes in response to TGOT (500 nm) + TTX (1 µm) in females. The vertical line indicates the 20s of TGOT application. (M). Proportion of LS astrocytes responding to TGOT application in male and female individuals (n_astro male LS_ = 63, n_record male LS_ = 14, n_astro female LS_ = 63, n_record female LS_ = 16, n_rat LS_ = 3–5). (N). Comparison of calcium activity triggered by TGOT application in male and female individuals. Spider plots showing the p‐value of the comparison between the basal state and after the application of TGOT for different calcium signaling parameters. (O). Bar plot showing mean frequency of calcium transients in male and females during baseline time (n_astro resp male LS_ = 14, n_record resp male LS_ = 14, n_astro resp female LS_ = 24, n_record resp female LS_ = 16, n_rat resp LS_ = 3–5). (P). Bar plots showing mean AUC and mean frequency of calcium transients triggered by TGOT application in male and females normalized to baseline values (n_astro resp male LS_ = 14, n_record resp male LS_ = 14, n_astro resp female LS_ = 24, n_record resp female LS_ = 16, n_rat resp LS_ = 3–5). (Q–X). OTR mRNA positive astrocytes in the rats CA2 and their response to OTR activation. (Q). High magnification confocal image shows overlap of S100b and OTR mRNA in the CA2 of a female rat. Scale bar = 10 µm (R). Dual immunostaining against GFAP and OTR protein reveals the presence of OTRs in somata and filaments of rat astrocytes within the CA2. Three‐dimensional reconstruction (images 3 and 4) demonstrates expression of OTR within GFAP‐positive processes. Scale bars = 100, 10, and 1 µm. (S). Heatmap of OGB1 fluorescence intensity of individual astrocytes in response to TGOT (500 nm) in female. The vertical line indicates 20s of TGOT application. (T). Typical ΔF traces of OGB1 fluorescence intensity of individual astrocytes in response to TGOT (500 nm) + TTX (1 µm) in females. The vertical line indicates 20s of TGOT application. (U). Proportion of CA2 astrocytes responding to TGOT application in male and female individuals (n_astro male CA2_ = 69, n_record male CA2_ = 15, n_astro female CA2_ = 114, n_record female CA2_ = 19, n_rat CA2_ = 3–5). (V). Comparison of calcium activity triggered by TGOT application in male and female individuals. Spider plots showing the p‐value of the comparison between the basal state and after the application of TGOT for different calcium signaling parameters. (W). Bar plot showing mean frequency of calcium transients in male and females during baseline time (n_astro resp male CA2_ = 26, n_record resp male CA2_ = 15, n_astro resp female CA2_ = 40, n_record resp female CA2_ = 19, n_rat resp CA2_ = 3–5). (X). Bar plots showing mean AUC and mean frequency of calcium transients triggered by TGOT application in males and females normalized to baseline values n_astro resp male (CA2_ = 26, n_record resp male CA2_ = 15, n_astro resp female CA2_ = 40, n_record resp female CA2_ = 19, n_rat resp CA2_ = 3–5). Data are expressed as mean across animal ± SEM. ^#^ *p* < 0.05, ^##^ *p* < 0.01, ^###^ *p* < 0,001 & ^*^ *p* < 0.05, ^**^ *p* < 0.01, ^***^ *p* < 0,001, two‐sided unpaired *t*‐test or Mann–Whitney U test or paired *t*‐test or Wilcoxon test. Detailed statistics can be found in Statistic Table S3.

Given that mRNA presence does not necessarily indicate functional protein expression [23, 26], we thought to perform a more detailed analysis in a selected number of brain regions in both male and female rats (CeA, CA2, LS, Figures 3 and S3, VMH, Figure S4, NAc, Figure 5). In all the analyzed brain regions, we detected substantial quantities of OTR mRNA (Figures 3A,I,Q, S4A, and 5A) and proteins, showing OTR protein expression in astrocyte somata and processes (Figures 3B,J,R , S4B, and 5B). To probe the functionality of detected OTRs, we performed ex vivo OGB1‐based calcium (Ca^2^^+^) imaging of astrocytes [27]. We bath‐applied the selective OTR agonist ([Thr^4^, Gly^7^]‐oxytocin, TGOT), and measured calcium transients in SR101‐identified astrocytes in brain slices in the presence of TTX to block action potential‐dependent neuronal activity (Figure 3). Overall in rats, TGOT elicited a response varying from 22.4% ± 7.8% to 63.0% ± 8.8% astrocytes (Figures 3E,M,U and 5E, Figure S4E) with no differences between sexes.

In the CeA (Figure 3A–H), the frequency of calcium transients of basal astrocytes’ calcium transients were similar in male and in female rats (Hz: ♀ 1.6 ± 0.6 vs. ♂ 2.6 ± 0.7, p = 0.1087; Figure 3G). Moreover, a similar proportion of astrocytes responded to TGOT application (♀ 52.7% ± 6.1% and ♂ 63.0% ± 8.8%, p = 0.3263, Figure 3E). This response was similar in both males and females in all parameters analyzed, including AUC frequency, height, width, and rise and decay time of calcium transients (Figure 3F,H and Figure S3C). Initially, astrocytic activity is not affected by sex, just as the effect of OT on astrocytic activity appears to be similar between males and females.

In the LS (Figure 3I–P), basal astrocytes calcium transients were significantly more frequent in females than in males (Hz: ♀ 4.5 ± 1.3 vs. ♂ 0.3 ± 0.3, p = 0.0029; Figure 3O). However, a similar proportion of astrocytes responded to TGOT application in females as in males (♀ 37.9% ± 8.4% and ♂ 22.4% ± 7.8%, p = 0.1422; Figure 3M). This response was similar in both males and females in all parameters analyzed, including AUC and frequency of calcium transients (Figure 3N,P and Figure S3F). OTR activation led to similar calcium transient responses in LS astrocytes of both sexes whereas basal astrocyte activity is more important in female.

In the hippocampal CA2 (Figure 3Q–X), basal astrocyte calcium transients were similar in frequencies (♀ 1.0 ± 0.4 vs. ♂ 0.7 ± 0.4 mHz, p = 0.8325; Figure 3W). Moreover, a similar proportion of astrocytes responded to TGOT application in both sexes (♀ 35.2% ± 5.5% and ♂ 38.4% ± 7.5%, p = 0.8290; Figure 3U). However, this response was similar in both males and females in all parameters analyzed (Figure 3V) except AUC. Indeed, astrocyte calcium transient ratios are higher in females (3.9 ± 0.4 AU) than in males (2.1 ± 0.3 AU, p = 0.0005; Figure 3X). Therefore, while no sex difference was detected in the basal activity of astrocytes, OTR activation produced kinetically different calcium responses in male vs. female CA2 astrocytes.

In the VMH (Figure S4), basal astrocyte calcium transients were not more frequent depending on sexes (Hz: ♀ 0.63 ± 0.24 vs. ♂ 0.89 ± 0.18, p = 0.3761; Figure S4G). Moreover, astrocytes in male rats do not respond in a higher proportion to TGOT than astrocytes of female rats (♀ 28.8% ± 2.3% and ♂ 34.8% ± 2.6%, p = 0.0526; Figure S4E), despite a significant difference in OTR mRNA expressing astrocytes (Figure 2F,G). This response was similar in both males and females in most of the parameters analyzed, with a faster decay time in calcium signal in females (Figure S4). Therefore, no sex difference was detected in the activity of astrocytes, OTR activation led to similar calcium transient responses in VMH astrocytes.

### 2.4 Anatomo‐Functional Analysis of OTR+ Astrocytes in the Mouse Brain

**FIGURE 4:**
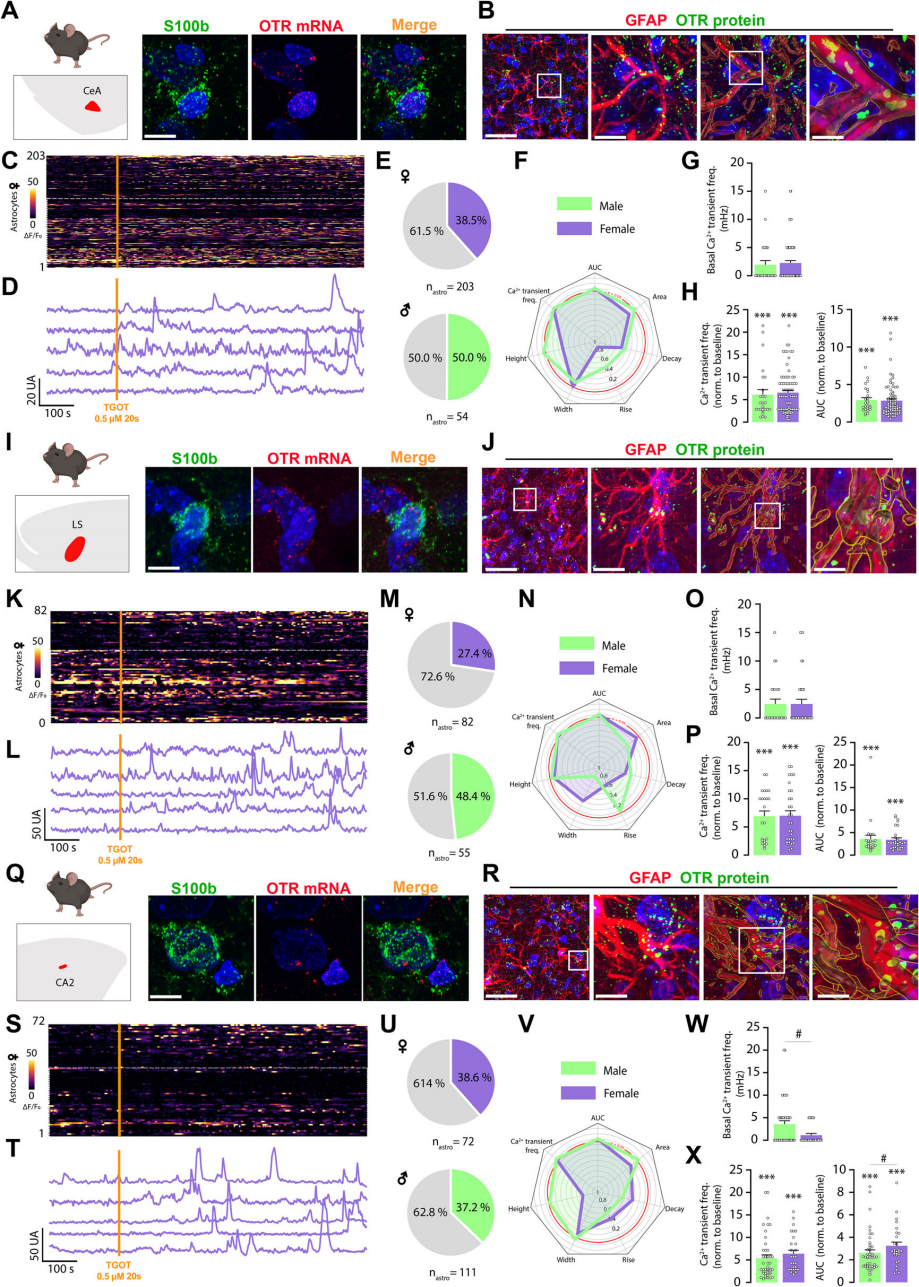
Anatomical mapping and functional assessment of OTR+ astrocytes in mouse CeA, LS and CA2. (A–H). OTR mRNA positive astrocytes in the mice CeA and their response to OTR activation. (A). High magnification confocal image shows overlap of S100b and OTR mRNA in the CeA of a female mouse. Scale bar = 10 µm. (B). Dual immunostaining against GFAP and OTR protein reveals the presence of OTRs in somata and filaments of mouse astrocytes within the CeA. Three‐dimensional reconstruction (images 3 and 4) demonstrates expression of OTR within GFAP‐positive processes. Scale bars = 100, 10, and 1 µm. (C). Heatmap of OGB1 fluorescence intensity of individual astrocytes in response to TGOT (500 nm) in females. The vertical line indicates 20s of TGOT application. (D). Typical ΔF traces of OGB1 fluorescence intensity of individual astrocytes in response to TGOT (500 nm) + TTX (1 µm) in females. The vertical line indicates 20s of TGOT application. (E). Proportion of CeA astrocytes responding to TGOT application in male and female individuals (n_astro male CeA_ = 54, n_record male CeA_ = 13, n_astro female CeA_ = 203, n_record female CeA_ = 16, n_mouse NAc_ = 3‐3). (F). Comparison of calcium activity triggered by TGOT application in male and female individuals. Spider plots showing the p‐value of the comparison between the basal state and after the application of TGOT for different calcium signaling parameters. (G). Bar plot showing mean frequency of calcium transients in male and females during baseline time (n_astro resp male CeA_ = 27, n_record resp male CeA_ = 13, n_astro resp female CeA_ = 78, n_record resp female CeA_ = 16, n_mouse NAc_ = 3‐3). (H). Bar plots showing mean AUC and mean frequency of calcium transients triggered by TGOT application in male and females normalized to baseline values (n_astro resp male CeA_ = 27, n_record resp male CeA_ = 13, n_astro resp female CeA_ = 78, n_record resp female CeA_ = 16, n_mouse NAc_ = 3‐3). (I–P). OTR mRNA positive astrocytes in the mice LS and their response to OTR activation. (I). High magnification confocal image shows overlap of S100b and OTR mRNA in the LS of a female mouse. Scale bar = 10 µm. (J). Dual immunohistochemical staining against GFAP and OTR protein reveals presence of OTRs in somata and filaments of mouse astrocytes within the LS. Three‐dimensional reconstruction (images 3 and 4) demonstrates expression of OTR within GFAP‐positive processes. Scale bars = 100, 10, and 1 µm. K. Heatmap of OGB1 fluorescence intensity of individual astrocytes in response to TGOT (500 nm) in females. The vertical line indicates 20s of TGOT application. (L). Typical ΔF traces of OGB1 fluorescence intensity of individual astrocytes in response to TGOT (500 nm) + TTX (1 µm) in female. The vertical line indicates 20s of TGOT application. (M). Proportion of LS astrocytes responding to TGOT application in male and female individuals (n_astro male LS_ = 55, n_record male LS_ = 14, n_astro female LS_ = 82, n_record female LS_ = 25, n_mouse LS_ = 4‐4). (N). Comparison of calcium activity triggered by TGOT application in male and female individuals. Spider plots showing the p‐value of the comparison between the basal state and after the application of TGOT for different calcium signaling parameters. (O). Bar plot showing mean frequency of calcium transients in male and females during baseline time (n_astro resp male LS_ = 27, n_record resp male LS_ = 14, n_astro resp female LS_ = 22, n_record resp female LS_ = 25, n_mouse LS_ = 4‐4).(P). Bar plots showing mean AUC and mean frequency of calcium transients triggered by TGOT application in male and females normalized to baseline values (n_astro resp male LS_ = 27, n_record resp male LS_ = 14, n_astro resp female LS_ = 22, n_record resp female LS_ = 25, n_mouse LS_ = 4‐4). (Q–X). OTR mRNA positive astrocytes in mice CA2 and their response to OTR activation. (Q). High magnification confocal image shows overlap of S100b and OTR mRNA in the CA2 of a female mouse. Scale bar = 10 µm (R). Dual immunostaining against GFAP and OTR protein reveals the presence of OTRs in somata and filaments of mouse astrocytes within the CA2. Three‐dimensional reconstruction (images 3 and 4) demonstrates expression of OTR within GFAP‐positive processes. Scale bars = 100, 10, and 1 µm. (S). Heatmap of OGB1 fluorescence intensity of individual astrocytes in response to TGOT (500 nm) in females. The vertical line indicates 20s of TGOT application. (T). Typical ΔF traces of OGB1 fluorescence intensity of individual astrocytes in response to TGOT (500 nm) + TTX (1 µm) in females. The vertical line indicates 20s of TGOT application in female. (U). Proportion of CA2 astrocytes responding to TGOT application in male and female individuals (n_astro male CA2_ = 111, n_record male CA2_ = 22, n_astro female CA2_ = 72, n_record female CA2_ = 10, n_mouse CA2_ = 3‐4). (V). Comparison of calcium activity triggered by TGOT application in male and female individuals. Spider plots showing the p‐value of the comparison between the basal state and after the application of TGOT for different calcium signaling parameters. (W). Bar plot showing mean frequency of calcium transients in male and females during baseline time (n_astro resp male CA2_ = 41, n_record resp male CA2_ = 22, n_astro resp female CA2_ = 28, n_record resp female CA2_ = 10, n_mouse CA2_ = 3‐4). (X). Bar plots showing mean AUC and mean frequency of calcium transients triggered by TGOT application in male and females normalized to baseline values (n_astro resp male CA2_ = 41, n_record resp male CA2_ = 22, n_astro resp female CA2_ = 28, n_record resp female CA2_ = 10, n_mouse CA2_ = 3‐4). Data are expressed as mean across animal ± SEM. ^#^ *p* < 0.05, ^##^ *p* < 0.01, ^###^ *p* < 0,001 & ^*^ *p* < 0.05, ^**^ *p* < 0.01, ^***^ *p* < 0,001, two‐sided unpaired *t*‐test or Mann–Whitney U test or paired *t*‐test or Wilcoxon test. Detailed statistics can be found in Statistic Table S4.

**FIGURE 5:**
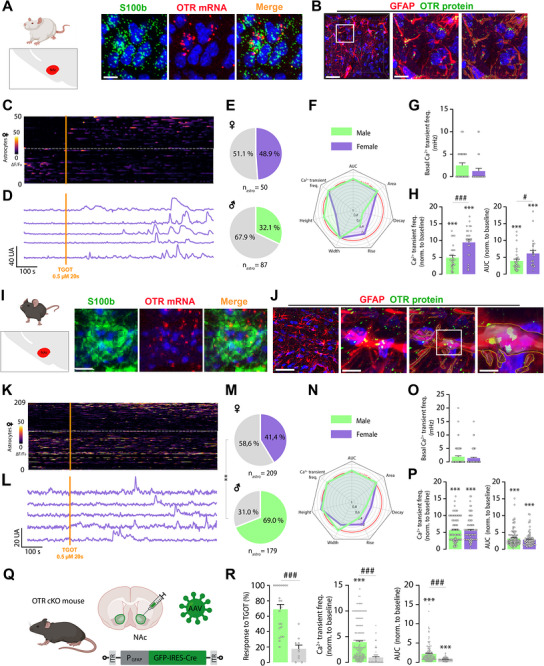
Mapping of the astrocytic OTR in rat and mouse NAc. (A). Schematic depiction of anatomical location of the rat NAc. Confocal images show a typical OTR+ astrocyte within the NAc. Scale bar = 10 µm. (B). Overview and high magnification of GFAP‐stained, OTR+ astrocyte in the rat NAc. Scale bars = 100 and 10 µm. (C). Heatmap of OGB1 fluorescence intensity of individual astrocytes in response to TGOT (500 nm) in female. The vertical line indicates 20s of TGOT application. (D). Typical ΔF traces of OGB1 fluorescence intensity of individual astrocytes in response to TGOT (500 nm) + TTX (1 µm) in female. The vertical line indicate the 20s of TGOT application. (E). Proportion of NAc astrocytes responding to TGOT application in male and female individuals (n_astro male NAc_ = 87, n_record male NAc_ = 27, n_astro female NAc_ = 50, n_record female NAc_ = 16, n_rat NAc_ = 3‐5). (F). Comparison of calcium activity triggered by TGOT application in male and female individuals. Spider plots showing the p‐value of the comparison between the basal state and after the application of TGOT for different calcium signaling parameters. (G). Bar plot showing mean frequency of calcium transients in male and females during baseline time (n_astro resp male NAc_ = 29, n_record resp male NAc_ = 27, n_astro resp female NAc_ = 23, n_record resp female NAc_ = 16, n_rat resp NAc_ = 3‐5). (H). Bar plots showing mean AUC and mean frequency of calcium transients triggered by TGOT application in male and females normalized to baseline values (n_astro resp male NAc_ = 29, n_record resp male NAc_ = 27, n_astro resp female NAc_ = 23, n_record resp female NAc_ = 16, n_rat resp NAc_ = 3‐5). (I). High magnification confocal image shows overlap of S100b and OTR mRNA in the NAc of a female mouse. Scale bar = 10 µm (J). Dual immunostaining against GFAP and OTR protein reveals presence of OTRs in somata and filaments of mouse astrocytes within the NAc. Three‐dimensional reconstruction (images 3 and 4) demonstrates expression of OTR within GFAP‐positive processes. Scale bars = 100 µm, 10 and 1 µm. (K). Heatmap of OGB1 fluorescence intensity of individual astrocytes in response to TGOT (500 nm) in female. The vertical line indicates 20s of TGOT application. (L). Typical ΔF traces of OGB1 fluorescence intensity of individual astrocytes in response to TGOT (500 nm) + TTX (1 µm) in female. The vertical line indicates 20s of TGOT application. (M). Proportion of NAc astrocytes responding to TGOT application in male and female individuals (n_astro male NAc_ = 179, n_record male NAc_ = 22, n_astro female NAc_ = 209, n_record female NAc_ = 16, n_mouse NAc_ = 5‐3). (N). Comparison of calcium activity triggered by TGOT application in male and female individuals. Spider plots showing the p‐value of the comparison between the basal state and after the application of TGOT for different calcium signaling parameters. (O). Bar plot showing mean frequency of calcium transients in males and females during baseline time (n_astro resp male NAc_ = 124, n_record resp male NAc_ = 22, n_astro resp female NAc_ = 87, n_record resp female NAc_ = 16, n_mouse NAc_ = 5‐3). (P). Bar plots showing mean AUC and mean frequency of calcium transients triggered by TGOT application in males and females normalized to baseline values (n_astro resp male NAc_ = 124, n_record resp male NAc_ = 22, n_astro resp female NAc_ = 87, n_record resp female NAc_ = 16, n_mouse NAc_ = 5‐3). (Q). Experimental strategy for the specific deletion of OTRs in mice NAc astrocytes (GFAP OTR cKO). (R). Proportion of NAc astrocytes responding to TGOT application in control (green) and cKO (dark grey) male individuals and bar plots showing mean AUC and mean frequency of calcium transients triggered by TGOT application in control and cKO male normalized to baseline values (n_astro male CTRL NAc_ = 179, n_record male NAc_ = 22, n_astro male cKO NAc_ = 118, n_record male cKO NAc_ = 10, n_mouse NAc_ = 5‐3). ^#^ *p* < 0.05, ^##^ *p* < 0.01, ### *p* < 0,001 & ^*^ *p* < 0.05, ^**^ *p* < 0.01, ^***^ *p* < 0,001, two‐sided unpaired *t*‐test or Mann–Whitney U test or paired *t*‐test or Wilcoxon test. Data are expressed as mean across animal ± SEM. Detailed statistics can be found in Statistic Table S5.

Based on the initial data acquired in rats, we were curious whether we could reproduce our data in selected brain regions in mice known for high OTR expression and their role in social and cognitive behavior (CeA, LS, CA2, Figure 4 and Figure S5, NAc, Figure 5 and Figure S6). In all the analyzed brain regions, we detected substantial quantities of OTR mRNA (Figure 4A,I,Q and, Figure 5I) finding that was confirmed by OTR immunostaining [18] showing OTR protein expression in astrocyte somata and processes (Figures 4A,I,Q and 5I). We then performed ex vivo OGB1‐based Ca^2^^+^ imaging of astrocytes [27] and analyzed the TGOT‐induced response. In mice, TGOT elicited a response in 27.4% ± 6.3% to 69.0% ± 6.3% astrocytes (Figures 4E,M,U and 5M). While this response appears similar between the sexes, the NAc marks an exception: 69.0% ± 6.3% astrocytes responded in males and only 41.4% ± 5.5% in females (Figure 5E; p = 0.0097).

Consistent with observations in rats, mouse CeA (Figure 4A–H), basal calcium transients’ frequencies are not different between males and females (Hz: ♀ 2.2 ± 0.4 vs. ♂ 1.9 ± 0.7, p = 0.5957; Figure 4G). By contrast, a similar proportion of astrocytes responded to TGOT application in both sexes (♀ 38.5% ± 4.4% % and ♂ 50.1 ± 6.4, p = 0.1364, Figure 4E). This response was similar in both males and females in all parameters analyzed, including AUC and frequencies of calcium transients (Figure 4F,H and Figure S5C). OTR activation led to similar calcium transient responses in CeA astrocytes of both sexes. Importantly, TGOT‐induced response was significantly decreased in presence of the OTR antagonist d(CH2)51, Tyr(Me)2, Thr4, Orn8, des‐Gly‐NH29)‐Vasotocin (dOVT; Figure S5D), indicating TGOT indeed recruit the OTR [11].

The same pattern of activity was observed in the LS (Figure 4I–P). Basal astrocytes calcium transients had a similar frequency in both sexes (Hz: ♀ 2.5 ± 0.8 vs. ♂ 2.5 ± 0.9, p = 0.8875; Figure 4O). While, a slightly higher proportion of astrocytes responded to TGOT application in males than in females (♀ 27.4% ± 6.3% and ♂ 48.4% ± 10.5%, p = 0.0942; Figure 4M). This response was similar in both males and females in most of the parameters analyzed, with a faster decay time in calcium signal in females (Figure 4N,P and Figure S5G).

In the CA2 (Figure 4Q–X), basal astrocyte calcium transients were more frequent in males than in females (♀ 1.1 ± 0.4 vs. ♂ 3.5 ± 0.8 mHz, p = 0.023; Figure 4W). However, a similar proportion of astrocytes responded to TGOT application (♀ 38.6% ± 5.4% and ♂ 37.2% ± 5.1%, p = 0.9913; Figure 4U). Moreover, as a sex difference was detected in astrocyte basal activity, OTR activation led to kinetically different calcium responses in male and female CA2 astrocytes. Indeed, astrocyte calcium transient AUC ratios are higher in females (3.2 ± 0,3 AU) than in males (2.6 ± 0.3 AU, p = 0.0342; Figure 3X). Here, sex difference was detected in the basal activity of astrocytes but also OTR activation produced kinetically different calcium responses between the two sexes in CA2 astrocyte.

Together, this detailed analysis of astrocytic calcium transients modulated by OTR activation in the rat and mouse brain highlights the functional heterogeneity and sexual dimorphism of astrocytic calcium activity.

### 2.5 Behavioral Involvement of OTR+ Astrocytes in the NAc

As previously observed in rats and mice in other regions, we observed colocalization between astrocytes markers and OTR mRNA (Figure 5I) as well as with the OTR protein itself (Figure 5J) within the NAc. To determine whether these receptors are functional, we again performed Ca^2^^+^ imaging in mouse and rat (Figure 5, Figures S6 and S7) brain slices.

In rats, frequencies of basal calcium transients were similar between both sexes (Hz: ♀ 1.3 ± 0.6 vs. ♂ 2.5 ± 0.6, p = 0.0717; Figure 5G). Also, the same proportion of astrocytes responded to TGOT in males and females (♀ 48.9% ± 7.6% and ♂ 32.1% ± 6.4%, p = 0.0728; Figure 5E). Interestingly, the TGOT‐evoked Ca^2+^ calcium response was bigger in females than in males (AUC ratio: ♀ 6.1 ± 0.9 vs. ♂ 3.9 ± 0.6 AU, p = 0.0158; Figure 5H) and displayed a higher frequency (Hz ratio: ♀ 9.5 ± 1.0 vs. ♂ 4.9 ± 0.6 mHz, p = 0.001; Figure 5H). No differences were detected in the remaining parameters (Figure S6C)

In mice, the basal astrocytes calcium transients had similar frequencies in both sexes (Hz: ♀ 1.4 ± 0.3 vs. ♂ 1.9 ± 0.3, p = 0.1989; Figure 5O). Interestingly, a larger proportion of astrocytes responded to TGOT application in males than in females mice (♀ 41.4% ± 5.5% and ♂ 69.0% ± 6.3%, p = 0.0097 Figure 5M), which was the larger proportion of astrocytes responding to TGOT measured across the tested brain areas. Furthermore, the TGOT‐evoked Ca^2+^ calcium response was similar between males and females (AUC ratio: ♀ 2.8 ± 0.2 vs. ♂ 3.2 ± 0.3 AU, p = 0.3792; Figure 5P) and also have the same frequencies (Hz ratio: ♀ 5.5 ± 0.4 vs. ♂ 5.6 ± 0.4 mHz, p = 0.9291; Figure 5P). No difference was detected in the other calcium transient parameters (Figure S6F). Therefore, a major sex difference was detected in the mice NAc, with male astrocytes being significantly more responsive to OTR activation.

**FIGURE 6:**
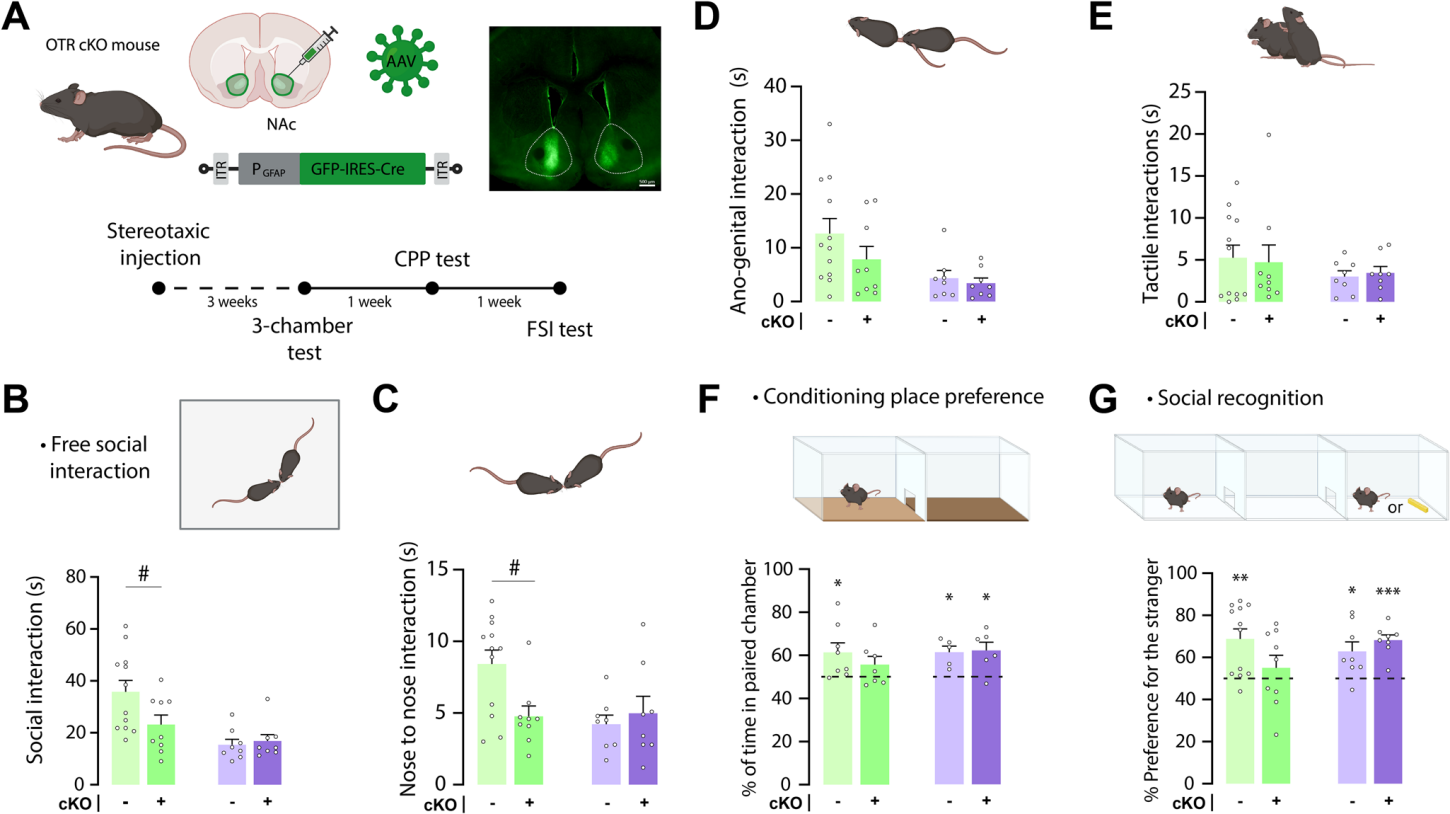
Involvement of NAc astrocytic OTR in mouse social behaviors. (A). Experimental strategy for the specific deletion of OTRs in mice NAc astrocytes (GFAP OTR KO) and behaviors tests. (B). Scheme of the experimental setups used for assessment of free social interaction test and bar plots showing mean active time with interactor during free social interaction tests spent by the dyad socially interacting (n_CTRL ♂_ = 12, n_OTR cKO ♂_ = 9, n_CTRL ♀_ = 8, n_OTR cKO ♀_ = 8). (C). Bar plot showing mean time of nose‐to‐nose interaction in male and female (n_CTRL ♂_ = 12, n_OTR cKO ♂_ = 9, n_CTRL ♀_ = 8, n_OTR cKO ♀_ = 8). (D). Bar plot showing mean time of ano‐genital interaction in male and female (n_CTRL ♂_ = 12, n_OTR cKO ♂_ = 9, n_CTRL ♀_ = 8, n_OTR cKO ♀_ = 8). (E). Bar plot showing mean time of others tactile interaction in male and female (n_CTRL ♂_ = 12, n_OTR cKO ♂_ = 9, n_CTRL ♀_ = 8, n_OTR cKO ♀_ = 8). (F). Scheme of the experimental setups used for assessment of the conditioning place preference test and bar plots showing mean time in paired chamber during conditioning place preference test (n_CTRL♂_ = 8, n_OTR cKO ♂_ = 7, n_CTRL ♀_ = 6, n_OTR cKO ♀_ = 6). (G). Scheme of the experimental setups used for assessment of social recognition test and bar plots showing mean percentage of preference for the stranger individuals during social recognition test (n_CTRL ♂_ = 12, n_OTR cKO ♂_ = 9, n_CTRL ♀_ = 8, n_OTR cKO ♀_ = 8). Data are expressed as mean across animal ± SEM. Detailed statistics can be found in Statistic Table S6. ^#^ *p* < 0.05, ^##^ *p* < 0.01, ^###^ *p* < 0,001 for SHAM vs. OTR cKo comparison with one way Anova & ^*^ *p* < 0.05, ^**^ *p* < 0.01, ^***^ *p* < 0,001, for one sample *t*‐test against chance (50%).

To directly assess the function of astrocytic OTR signaling in the NAc, we induced astrocyte‐brain region‐specific ablation of OTRs. To this end, we injected an AAV encoding the Cre recombinase under the control of the GFAP promoter (rAAV‐GFAP‐Cre‐GFP) in the NAc of OTR fl/fl mice (GFAP OTR cKO, Figure 6A) [12, 13]. Control animals were OTR fl/fl mice injected with an inert virus (rAAV‐GFP). The specificity and efficacy of the astrocyte specific OTR deletion were assessed with multiplexed RNAscope for Aldh1 and OTR mRNA, as well as calcium imaging (Figure S7). Importantly, calcium imaging supports the functional deletion of astrocytic OTR, almost ablating the TGOT‐evoked response (Figure 5Q,R and Figure S6G–H).

The NAc has been shown to play a key role in mediating social reward and novelty, with OT signaling enhancing the salience of social stimuli [20]. OT action in this region is also critical for social recognition, as disrupting its signaling impairs the ability of mice to distinguish familiar from novel conspecifics [21]. Therefore, the previously detected difference in OTR astrocytes responses to TGOT in males and females led us to take a closer look at astrocytic OTRs in NAc to determine their potential role in social behavior. To do so, three weeks after viruses injections, we tested mice for social behaviors [28] (Figure 6 and Figure S8).

We evaluated involvement of NAc astrocytic OTR in a free social interaction test, where the test mouse (SHAM or NAc OTR cKO) freely interacted with a same‐sex naïve conspecific during 5 min (Figure 6B–E and Figure S8A,B). Interestingly, dyads with male NAc OTR cKO mice showed decreased social investigation levels than controls (p = 0.0469; Figure 6B), an effect that was not observed in females. This decrease in social investigation was mainly explained by a reduced time spent in direct nose‐to‐nose investigation (p = 0.0146; Figure 6C), whereas no significant differences were found in other types of social interaction such as ano‐genital investigation (Figure 6D), tactile interactions (Figure 6E) or pursuits (Figure S8A). This indicates that NAc astrocytic OTRs modulate engagement in dynamic social interactions in male mice.

We further explored the involvement of NAc astrocytic OTR in social memory and performed a social place‐preference conditioning test (Figure 6F). Each animal was first conditioned during 24 h to a group‐housing litter (social bedding). On the next day, the same animal was exposed 24 h to a solo‐housing clean litter (neutral bedding). On the third day, animals were given the choice to explore both beddings in a 2‐chamber box. As expected, all control animals, males (p = 0.035) and females (p = 0.0159), did show preference for the social bedding. However, only the NAc OTR cKO female mice (p = 0.0255) preferred the social bedding, while the NAc OTR cKO male mice showed no preference for either beddings (p = 0.1871). No difference was observed in total distance traveled (Figure S8C). These results suggest that NAc astrocytic OTRs in males might have a role in social memory or in the rewarding properties of social interactions.

To disentangle this, we performed a social recognition test (Figure 6G). When testing animal's preference for a new same‐sex conspecific individual over the familiar mouse, all control animals, both males (p = 0.0049) and females (p = 0.0242), spent more time exploring the new conspecific (Figure 6G and Figure S8G). However, only the female NAc OTR cKO presented such preference (p = 0.0002), while males showed no preference for the novel conspecific (p = 0.4300). No difference was observed in total distance traveled (Figure S8F). These results indicate that NAc astrocytic OTRs in males are involved in the recognition of new conspecific individuals.

Collectively, these data indicate a crucial role of male NAc astrocytic OTRs in active social interactions and social memory.

## 3 Discussion

Here, we provide a widespread anatomical and functional analysis of OTR+ astrocytes in the mouse and rat brain. Our comprehensive characterization using RNAscope in situ hybridization, immunohistochemical labeling, and calcium imaging revealed that OTR+ astrocytes are widely distributed and display distinct molecular and functional properties. Sex differences in NAc OTR+ astrocytes activity led us to investigate their function and identify their specific involvement in social recognition and active social interactions for male mice.

This novel systematic atlas not only provides a comprehensive framework for understanding the spatial organization of OT signaling but also sheds light on the potential brain region‐specific roles of astrocytes in mediating the diverse physiological and behavioral effects of OT. Our combined anatomical, functional, and behavioral data indicates a crucial physiological role of OTR+ astrocytes across the brain. These findings provide a new rationale for the dynamic and precise OT‐ergic modulation of brain circuits through targeted activation of astrocytic OTRs. Moreover, this suggests that OTR+ astrocytes are important players in OT‐ergic signaling, not merely in select regions but throughout the entire brain.

We employed a combinatory approach using RNAscope‐based mRNA detection and immunohistochemistry to identify and quantify OTR+ astrocytes throughout the brain. GFAP is a widely used but imperfect astrocyte marker [23], as many astrocytes show low baseline expression under healthy conditions, and GFAP staining captures only ∼15%–20% of the astrocyte primarily cytoskeletal processes [29]. Thus, we potentially undersampled filamentous OTRs given that we were only able to visualize main cytoskeletal processes with GFAP staining. To address potential limitations, we cross‐validated our pipeline with complementary approaches using Aldh1 and GS (Figure 2), which confirmed the robustness of our findings. While protease treatment, a necessary step for RNAscope, inevitably reduces astrocyte signal intensity, we were still able to reliably detect OTR+ astrocytes across multiple regions of the mouse and rat brain. Our comprehensive analysis of OTR+ astrocytes in the mouse and rat brain offers a pivotal resource for the scientific community, but comes with several limitations: A major limitation of the astrocytic OTR mapping experiments is the relatively sparse sampling per region (2‐6 sections per animal), which may not fully capture intra‐regional variability in OTR+ astrocyte distribution; accordingly, the reported patterns should be interpreted as representative rather than exhaustive, and future studies with denser sampling will be needed to refine regional estimates. By detailing the distribution and abundance of OTR+ astrocytes across various brain regions, our atlas enables researchers to investigate how OT signaling influences regional brain functions. It supports targeted investigations into the cellular mechanisms underlying OT's diverse effects on brain function and behavior. Astrocytic OTRs play a pivotal role in modulating neuronal networks and behaviors by influencing astrocyte signaling pathways and neuron‐astrocyte interactions [12, 14]. Within the CeA, activation of OTRs in astrocytes has been shown to induce cytoskeletal remodeling [13, 14], thereby affecting intercellular connectivity within neuronal circuits. Additionally, OT‐mediated signaling in astrocytes can modulate synaptic transmission, contributing to the regulation of stress responses and anxiolysis [16]. Together, these findings suggest that astrocytes, through OT signaling, are integral to the neuromodulation of emotional and social behaviors.

Several studies in the last few years have convincingly demonstrated the importance of astrocytic OTR signaling in the modulation of neuronal activity, physiology, and behavior as well as their relevance in cardiovascular disease and neurodevelopmental disorders [11, 12, 14, 18, 30, 31, 32]. Thus, a fundamental question remains: If astrocytic OTRs are so critical for the initial response to released OT, what exactly is the role of extrahypothalamic neuronal OTRs? As previously proposed [14], it seems plausible that OT acts in a two‐pronged approach: A fast activation of astrocytic OTRs and subsequent release of gliotransmitters, and a slower response through neuronal OTRs that facilitate a long‐lasting effect, thus keeping neurons longer near to threshold potential, allowing the generation of action potentials with minimal stimuli.

Remarkably, both OTR‐Gαi and OTR‐Gαq pathway activation lead to Ca^2+^ transients in astrocytes, yet with distinct signatures [33]. How do astrocytes differentiate between these two signaling pathways to trigger an appropriate reaction? Interestingly, the presence of OTR mRNA within astrocytic processes suggests that OTR expression may be regulated locally, enabling rapid, spatially restricted responses to synaptic activity or neuromodulatory cues. Local translation in astrocyte processes has been increasingly recognized as a mechanism for fine‐tuning receptor availability and signaling dynamics close to sites of neuron‐glia interaction [34, 35]. Therefore, such compartmentalized control could allow astrocytes to modulate OT signaling with different intracellular pathway‐Gαi or Gαq depending on the subcellular location of the OTR. Moreover, it would allow an OT‐mediated high temporal and spatial precision, potentially influencing synaptic transmission, plasticity, or neuroendocrine feedback. This localized regulation might therefore represent an additional layer of complexity in OTR‐mediated astrocyte‐neuron communication.

OT signaling within the NAc is integral to modulating social behaviors and social rewards [20]. In prairie voles, for instance, OTR activity in the NAc facilitates social reward and pair bonding, with disruptions impairing these behaviors [19, 36]. Additionally, OT in the NAc core induces long‐term depression of excitatory synaptic transmission in medium spiny neurons, serving as a social reinforcement signal. Taken together, our behavioral assays, led to reveal a contribution of NAc astrocytic OTRs in social recognition and specifically nose‐to‐nose interactions. The nose‐to‐nose interaction is a non‐aversive and specific to a pro‐social pattern, with mice more likely to let themselves be approached by the front part of their body than by the posterior parts [37, 38, 39]. This suggests that NAc astrocytic OTRs in males primarily regulate pro‐social affiliation during direct social interaction rather than the rewarding value of social contact per se. Further research is necessary to elucidate the specific contributions of astrocytic OTR signaling in the NAc core and shell subregions to social behaviors and rewards. However, one should consider that null findings in the behavioral assays performed should be interpreted cautiously given the inherent variability of behavioral data.

Importantly, our analysis revealed several sex differences in OTR expression and function in astrocytes. OTR binding and expression in the VMH is modulated by both testosterone and estrogen, indicating a hormonal influence on receptor expression [40, 41], highlighting a sex‐specific regulation of OT signaling. Notably, we observed drastic sex differences in term of astrocytic function within the NAc, in both calcium signaling and behavior, with no detected difference in OTR mRNA levels. This indicates two findings. First, the level of expression of a neuropeptidergic receptor may be largely disconnected from its functional importance, depending on the cellular network involved. The role of astrocyte OTR‐mediated modulation might be the fine‐tuning of neuronal networks, allowing their correct function and subsequent behavioral modulation, as suggested by a series of manuscripts recently published [42, 43, 44]. Second, the dissociation between receptor expression and functional output suggests sex‐specific circuit integration or neuromodulatory context, consistent with prior work showing sexually dimorphic oxytocin signaling in the NAc and its role in social behaviors [20, 45]. Together, our findings extend this literature by identifying astrocytes as a previously unrecognized, sex‐specific substrate of oxytocinergic modulation in the NAc of affiliative interactions.

In our study, we noted only an impairment of nose‐to‐nose contacts between males, suggesting a specific function of NAc astrocytic OTR in in social recognition and active social interactions of males. One possible reason we failed to detect an effect in females is that our data was acquired from virgins. Indeed, it was previously shown that OTR function is plastic and largely depends on the life status of the female, from virgin to pregnant or lactating [46, 47, 48]. Therefore, it would be interesting to investigate if the involvement of NAc astrocytic OTRs in females changes over the reproductive cycle.

In conclusion, our present work constitutes a framework for further studies investigating the OT system, highlighting the critical presence and function of astrocytes in OT‐based modulation of brain circuits and functions. Our brain‐wide analysis of OTR‐expressing astrocytes reveals a previously unrecognized, widespread glial mechanism underlying OT signaling, with significant relevance for social behavior regulation. Future studies should aim to dissect the precise cellular and circuit mechanisms by which astrocytic OT receptor signaling influences physiology and behaviors, for example through cell‐type–specific manipulations combined with in vivo calcium imaging or electrophysiology. Such experiments could clarify how astrocyte‐neuron interactions contribute to social deficits and whether restoring astrocytic OT signaling can normalize circuit function. In a translational context, these insights may open new therapeutic avenues for autism spectrum disorders (ASD), where altered OT pathways [49] and glial dysfunction [50, 51] have both been implicated. Ultimately, understanding the astrocytic component of OT signaling could help refine targeted, brain‐region–specific strategies for modulating social behavior. Finally, by demonstrating a critical, sex‐specific role of NAc astrocytic OTRs in in social recognition and active social interactions, our study highlights novel therapeutic cellular targets beyond neurons for precision medicine approaches in social dysfunction.

## 4 Methods

### 4.1 Animals

Animals were housed under standard conditions with food and water available ad libitum and maintained on a 12‐h light/dark cycle and housed in groups of 3 to 5 under standard conditions with enrichment: regular poplar sawdust, cardboard curl, wood stick and tunnel per cage. All experiments were conducted in accordance with European Union rules and approbation from the French Ministry of Research (APAFIS #34228‐2021111710469619 v18; APAFIS #15541‐2018061412017327 v5, APAFIS #46161‐2024011918045241 v2, APAFIS #50278‐2024070216433172 v14) and German Ethical Committee (T‐37/23 and T‐33/23). For ex vivo experiments, male and female Sprague Dawley rats or C57BL/6J mice were used. For in vivo experiments, male and female OTR‐floxed mice were used. Ex vivo experiments used animals between two and six months old at the time of sacrifice. For in vivo experiments, animals that were six weeks old at the time of the first surgery were used.

### 4.2 Stereotaxic Surgery: Injection of rAAV Vectors

Stereotaxic surgery was performed on mice anesthetized with 1.5%–2% isoflurane and receiving Metacam (i.p, 10 mg/kg), Bupivacaine (s.c., 2 mg/kg) and lidocaine (s.c., 2 mg/kg) applied locally. AAV were injected into the NAc and allowed to express for 3 weeks. For specific deletion of OTR in mice NAc astrocytes, 300 nL of rAAV serotype ½ (GFAPp‐GFP‐IRES‐Cre) was injected bilaterally at the coordinates corresponding to NAc:ML:± 0.75 mm; AP:+1.7 mm; DV: −4.5 mm (according to Paxinos and Watson Brain Atlas) in OTR floxed mice. Any animal that received an injection outside the region concerned was removed from the analyzed data.

#### 4.2.1 Specific Deletion of OTRs in NAc Astrocytes

To specifically ablate OTRs in NAc astrocytes, transgenic cKO mice, in which loxP sites flank the OTR coding sequence18, received bilateral injections (300 nL) of rAAV‐GFAP‐GFP‐IRES‐Cre. For validation and references, [12, 13]. After 4 weeks of expression of the viral proteins, mice were intracardially perfused with 1× PBS and 4% paraformaldehyde (PFA).

### 4.3 Perfusion and Combinatory Immunohistochemistry and RNAscope Procedures

Perfusion was performed using a peristaltic pump to ensure constant flow rates of PBS and PFA. Chilled PBS (100 mL for rats, 10 mL for mice) was used, followed by 4% paraformaldehyde (PFA, 150 mL for rats, 10 mL for mice) for fixation. The perfusion rate was adjusted to 10–15 mL/min for rats and 5–6 mL/min for mice. Once completed, the brain was carefully extracted by cutting the optic tract and removing the skull, ensuring minimal tissue damage. Post‐fixation was carried out in 4% PFA for 4 h at 4°C, followed by dehydration in 30% sucrose in PBS until the brain sank (typically 36–48 h). Brains were then wrapped in aluminum foil and stored at ‐80°C. The combined IHC/RNAscope procedure was performed as previously described [25]. To enhance antigen accessibility, the sections underwent protease treatment before mRNA probe incubation. RNAscope in situ hybridization was used to detect OT receptor (OTR) mRNA. The procedure included protease treatment to enhance probe penetration, followed by hybridization with an OTR‐specific probe at 40°C for 2 h. Signal amplification was carried out through sequential incubations with AMP1‐3 and HRP‐conjugated reagents. Opal/TSA Plus fluorophores were then applied to visualize the hybridized mRNA. A final HRP blocking step was included to prevent nonspecific binding. Negative and positive control probes were used in parallel to validate specificity. Following RNAscope, sections were processed for immunohistochemistry, allowing co‐localization of OTR mRNA with astrocyte markers (GFAP, GS and S100b). For immunohistochemistry, brain sections were incubated with primary antibodies against GFAP, GS and S100b at dilutions of 1:250 or 1:500, OXTR (Alomone labs, AVR‐0013) was used at 1:100. Following overnight incubation at 4°C with primary antibodies, sections were washed and incubated with secondary antibodies conjugated to Alexa 488, Alexa 594, or Alexa 647 for 4 h at room temperature. No additional blocking was required after RNAscope. Sections were then mounted with an antifade medium containing DAPI, coverslipped, and sealed with nail polish to prevent drying.

### 4.4 Image Acquisition and Imaris‐Based Pipeline for OTR+ Astrocyte Quantification

Using the SlideScanner Olympus VS200, we captured overview images of whole brain slices with channels consisting of DAPI (405 nm), OTR mRNA (TSA Vivid 650), and GFAP mRNA (TSA Vivid 520). Slices were automatically recognized and manually adjusted, with focus points centered on even surfaces of the brain slice to avoid partial focus on ventricles. Three spot functions were generated to detect values above a set threshold for DAPI (min. 51.8), OTR‐mRNA (min. 20), and GFAP‐mRNA (min. 21.1). Images were converted from VSI files into Imaris files, and the spot functions were applied. The files were opened in Imaris, and the overview image was aligned with the Rat Brain Atlas using stereotaxic coordinates. If slices were tilted, multiple reference images were used for alignment, with spot functions deactivated during this process. After alignment, surfaces for regions of interest were created using the Surface tool, with contours drawn and named according to visible regions. Brain regions were defined as follows: S1 (S1, S1BF, S1DZ, S1FL, S1HL, S1DZO, S1Sh, S1Tr, S1ULp), S2 (S2), Pir (DEn, Apir, IEn, Pir, Pir1, REn), IC (AI, AID, AIP, AIV, DI, GI), ILC (IL), Hyp (SO, PVN, AHO, DA, PaMP, PaPo, AHC, PaMM, PaV, PaMP, AHA, LA, Pe, SPa, VLH, PLH, MPA, JPLH, PaAP, Spa, MPO, ESO, VLH, LA, RCh, MPOM, SChDL, SChVM, MPOL), CeA (CeC, CeL, CeM), BLA (BLA, BLP, BMP, BLV, BMA), dorsal CA2 (CA2 including neighboring areas within a 500 µm radius), VTA (VTAR), LS (LSI [1, 2, 3, 4, 5], LSD, LSV, PLd, SFi, fi, TS, f), NAC (AcbC, aca [partially if within AcbC], STMA), NAS (AcbSh, possibly ICjM, STMA), PAG (DMPAG, DLPAG, LPAG, VLPAG, PIPAG, p1PAG, Lth, SCO, A11), VMHyp (VMHDM, VMHC, VMHVL, VMHSh, VMH), AO (AOM, AOD, AOL, AOVP, AOP), DTT (DTT 1–3), NBM (NBM), SHy (SHy), mfba (MCPO/mfba), aa (AA), PV (PVA, PV, PVP, not taken when split into two separate regions), and ZI (most medial part of ZIV, most medial part of ZID, A13, possibly partially PaXi). In the edit window of each surface, the mask option was applied to Channel 1 (DAPI), and new channels were renamed based on the regions of interest. The DAPI spot function was activated, and the “Intensity Max Channel” filter was applied to the newly generated channels. New spot functions were created to isolate DAPI spots within the regions of interest and further filtered using the “Shortest Distance Spots” filter with a 10 µm threshold relative to GFAP spots. New spot functions were generated based on this filter for each region. The OTR channel was masked using the OTR spot function to isolate signals above the threshold, and the “Intensity Max Channel” filter was applied to the final spot function for regions of interest. The resulting bimodal graph was analyzed, with the left peak representing non‐overlapping spots and the right peak representing overlapping spots, which were documented in the labels window. For confocal imaging, a series of z‐stack images were taken using the Leica Stellaris 5 microscope at 40x magnification with a glycerol immersion objective. Images were taken for the first set of regions using 1 × 1 to 3 × 3 squares in 1 µm z‐stacks per animal, with approximately two images per region across all slides. The second set of regions was imaged using a 1 × 1 square, with each region sampled twice per animal. For rats, DAPI was imaged alongside TSA Vivid 520, 550, and 650. The first imaging session took place one to two days after IHC completion, while the second session was conducted nearly two weeks later. Technical imaging parameters were as follows: image dimensions of 1024 × 1024, bidirectional scan direction, scan speed of 400 Hz, pinhole size of 1.00 AU, zoom of 1.00, and voxel size of 0.285 µm × 0.285 µm × 1.00 µm. Leica (LIF) confocal files were converted into Imaris files, and three functions were used for analysis: one surface for reconstructing the DAPI signal, one for GFAP protein, and one spot function for OTR mRNA. The following thresholds were applied: DAPI (min. 11), GFAP (min. 13), and OTR (auto‐creation by Imaris, typically 1. For merged or stitched images, the DAPI surface was manually filtered for “Overlap Surface = Surface GFAP” to create a new surface of all nuclei overlapping with GFAP. This surface was manually cleared of noise artifacts, such as blood vessels and bleach‐throughs. The OTR spot function was then masked using the OTR channel to create a new channel, which was used in a new surface filtered with “Intensity Channel Max.” The surface was then relabeled accordingly. Astrocytes were classified as OTR+ if at least two distinct mRNA puncta within 2 µm of a DAPI‐stained nucleus were detected. All data were exported as.xls files and compiled using custom‐written Python scripts.

#### 4.4.1 Tissue Fixation

Animals were anesthetized using ketamine (Ketamin 1000, 400 mg/kg) and xylazine (Paxman, 80 mg/kg) administered intraperitoneally. Animals were then perfused transcardially with PBS, followed by 4% PFA. Brains were dissected and post‐fixed overnight in 4% PFA at 4°C.

### 4.5 Ex Vivo Calcium Imaging

#### 4.5.1 Brain Slices Preparation

Animals were anaesthetized using a mix of ketamine (Imalgene, i.p., 100 mg/kg) and xylazine (Rompun, i.p., 20 mg/kg). Lidocaine (s.c., 10 mg/kg) was administered locally. Intracardiac perfusion was then performed using one of the following artificial cerebrospinal fluid (aCSF) dissection solutions. For animals between 2 and 6 months old, an ice‐cold NMDG‐based aCSF was used containing (in mM): NMDG (93), KCl (2.5), NaH2PO4 (1.25), NaHCO3 (30), MgSO4 (10), CaCl2 (0.5), HEPES (20), D‐glucose (25), L‐ascorbic acid (5), thiourea (2), sodium pyruvate (3), N‐acetyl‐L‐cysteine (10) and kynurenic acid (2). After decapitation, the brain was swiftly removed in the same ice‐cold dissection aCSF used for intracardiac perfusion, and 350‐µm‐thick horizontal or coronal slices containing the CeA, CA2, VMH, PFC, NAc, and LS was obtained using a Leica VT1000S vibratome. Upon slicing, brain slices were hemisected and placed in a holding chamber at room temperature containing normal aCSF for 1 h minimum before any experiments were conducted. aCSF osmolality was between 290 and 310 mOsm/L aCSF pH was adjusted to 7.3–7.4 using either NaOH or HCl after bubbling in 95% O_2_/5% CO_2_ gas. Bubbling was maintained throughout the duration of the experiment. Also, all, in calcium imaging experiments, slices were transferred from the holding chamber to an immersion recording chamber and superfused at a rate of 2 mL/min with normal aCSF, unless indicated otherwise.

#### 4.5.2 Drug Application

OTR agonist [Thr4Gly7]oxytocin (TGOT, 0.5 µm) was bath or puff applied through a 20‐s‐long pumping of agonist solution, corresponding to several times the volume of the recording chamber. Other drugs (TTX, 1 µm) were applied for at least 20 min in the bath before performing any experiments. OTR antagonist d(CH2)51, Tyr(Me)2, Thr4, Orn8, des‐Gly‐NH29)‐Vasotocin (dOVT, 1 µm) and V1aR antagonist (SR49059, 10 nm) were also applied during negative control experiments for at least 20 min in the bath before running any experiments.

#### 4.5.3 Data Acquisition

To identify astrocytes, SR101 (1 µm) was added to aCSF in a culture well, and slices were incubated for 20 min at 37°C. After SR101 incubation, the synthetic calcium indicator OGB1‐AM was bulk loaded as previously described [51], reaching final concentrations of 0.0025% (20 µm) for OGB1‐AM, 0.002% for Cremophor EL, 0.01% for Pluronic F‐127 and 0.5% for DMSO in aCSF and incubated for 40min‐1 h at 37°C Slices were then washed in aCSF for at least 1 h before imaging. Only astrocytes co‐labeled for SR101 and OGB1 were used. The spinning disk confocal microscope for calcium imaging was composed of a Zeiss Axio examiner microscope with 20x and 40x water immersion objectives (numerical aperture of 1.0), mounted with an X‐Light Confocal Unit–CRESTOPT spinning disk. Images were acquired at 2 Hz with an optiMOS sCMOS camera (Qimaging). Cells within a confocal plane were illuminated for 20 ms at 575 nm for SR101 and 80 ms at 475 nm for OGB1 using a Spectra 7 LUMENCOR. The different hardware elements were synchronized through the MetaFluor 7.8.8.0 software (Molecular Devices). Because astrocytes are mechanosensitive, OTR agonists (500 nm) were bath applied for 20s and not puff‐applied to avoid mechanical stimulation. However, control experiment with puff application of TGOT can be find in Figure S6H. All calcium imaging experiments were conducted at 22°C room temperature and cells with an unstable baseline were discarded.

#### 4.5.4 Data Analysis

All analyses were conducted as described previously [27]. Astrocytic calcium levels were measured in manually outlined regions of interest (ROI) comprising the cell body using ImageJ software. Subsequent offline data analysis was performed using a custom‐written Python‐based script. To correct for small tissue movements that can introduce artifacts into the fluorescence signal, the SR101 fluorescence (motion reference) was subtracted from the OGB1 fluorescence (calcium signal). Then, a linear regression was applied to each trace to correct for photobleaching. Calcium transients were detected using the find peaks function of the SciPy library as fluorescence variation exceeding 8 time the standard deviation and a prominence exceeding it five times (for more details, see [27]. The number of peaks and the area under the curve was quantified before and after the drug application. All data were normalized according to the duration of the recording, and astrocytes were labeled as ‘responsive’ when their AUC or their calcium transient frequency at least doubled after drug application. Because the time after stimulation is longer than the baseline (11.40 min vs. 3.20 min), the probability of observing a spontaneous calcium peak is stronger after stimulation. To avoid this bias, astrocytes with only one calcium peak during the whole recording were not considered responsive. Each calcium transient was then isolated, its area was estimated with the trapezoid method, and its duration was measured as its half‐maximum full width (HMFW). The rise and decay phases of Ca^2+^ transients were fitted with linear functions and the coefficient of these slopes was used as the rise and decay constants. Fiji software was also used on SR101/OGB1 pictures to produce illustrative pictures. All calcium imaging experiments were conducted at controlled room temperature (22°C), and cells with an unstable baseline were discarded.

### 4.6 Behavioral Analysis

#### 4.6.1 Social Conditioned Place Preference

The device was composed of four soundproof boxes (56 × 56 × 43 cm), each containing a two‐chamber arena (40 × 20 × 28.5 cm; Omnitech Electronics, USA) equipped with infrared beams and a software interface to monitor mouse position. The two compartments differed by visual wall patterns (vertical vs. horizontal black‐and‐white stripes) and bedding type. The left compartment contained cellulose squares (Alphadry, Serlab; sawdust A), whereas the right compartment was covered with a mixed bedding (sawdust B) composed of 25% softwood pellet sawdust (Labsorb, Serlafe) and 75% irregularly cut poplar sawdust (Apsen Mix, Serlab).

After habituation to handling with a tunnel, the procedure was carried out over three days. On Day 1, a 30‐min pre‐test was performed to ensure no initial preference between compartments. The apparatus was cleaned with disinfectant (Surfa'Safe, Anios Laboratory) after each trial. Following this session, animals were returned to group housing for ∼24 h with sawdust A (conditioning 1). They were then placed in individual housing for ∼24 h with sawdust B (conditioning 2). Finally, on Day three, animals were reintroduced into the two‐compartment arena containing both types of sawdust for a 30‐min test. This post‐conditioning trial was used to assess preference for the two conditioned cues.

Conditioned place preference (CPP) was defined as a learned association between a condition (e.g., social context) and a contextual cue (e.g., bedding). Exclusion criteria were strictly defined as a pre‐conditioning preference score >1.5 or <0.5. Differences between pre‐ and post‐conditioning preference scores were considered significant when paired Student's *t*‐test p values were <0.05. In addition, one‐sample *t*‐tests against chance level (50%) were performed on the percentage of time spent in each compartment: $\%\;time\;compartiment=100*(\frac{time\;in\;compartiment}{1800s})$

#### 4.6.2 Social Interaction Test

Interactions between a test subject and an interactor mouse were manually scored and videotaped for 5 min. Light intensity was set at 30 lux. All animals were brought to the testing room 30 min prior to acclimate. The test and interactor mice were then simultaneously introduced into a clean cage (32 × 16 × 14.5 cm) containing a thin layer of bedding. Interactor mice were of the same C57BL/6J background, sex, and age, and were used at most twice per day. The order of testing was counterbalanced according to genotype and stimulus mice, and the experimenter was blinded to condition. The duration of social behaviors displayed by the test subject: facial sniffing (nose‐to‐nose contact), anogenital sniffing (nose‐to‐base sniffing), direct paw contact, and pursuit of the interactor mouse, and the social investigations by the interactor mice that were not reciprocated by the test mouse were recorded in real time using an ethological keyboard (ANY‐maze software). After testing, each mouse was returned to its home cage, and a new clean cage was used for the subsequent trial.

#### 4.6.3 Three Chambers Test

The apparatus (61.5 × 40 × 22 cm) consisted of three chambers, each 20 cm wide, separated by sliding doors (5 × 5 cm). Behavioral testing for sociability and social novelty preference was conducted using this setup. During a 2‐min habituation period, a subject mouse was placed with a familiar handling tunnel in the central chamber. The sliding doors were then opened, allowing the mouse free access to the entire arena for 5 min. Location and activity were automatically recorded with ANY‐maze video tracking software. Following this habituation phase, the mouse was returned to the central chamber and the doors were closed.

For the sociability test (Phase 1), a single unfamiliar C57BL/6J mouse was placed inside a small wire cage in one of the outer chambers, while a toy (Lego) was placed inside an identical wire cage in the opposite chamber. The sliding doors were then opened, and the subject was allowed to explore both stimuli for 5 min. Exploration behavior, defined as sniffing or touching the stimulus, was manually scored, while the time spent in each chamber was automatically recorded with ANY‐maze. To avoid side bias, the placement of the stimulus mouse was alternated between trials.

For the social novelty test (Phase 2), the subject was again placed in the central chamber with the doors closed. The familiar mouse from Phase 1 remained in its cage, whereas the toy was replaced by a novel unfamiliar mouse. The sliding doors were opened, and the subject's exploration behavior and location were recorded for another 5‐min session.

Stimulus mice had been habituated to being inside the wire cages (diameter: 8 cm, height: 10 cm) in two prior 5‐min sessions.

The percentage of exploration time directed toward each stimulus (toy or mouse) was calculated over the 5‐min test period:

$$\begin{array}{cc} & \%\;\mathrm{expl}\mathrm{orat}\mathrm{ion}\;\mathrm{per}\;\mathrm{stim}\mathrm{ulus}\\ & \;=100*\left(\frac{\mathrm{expl}\mathrm{orat}\mathrm{ion}\;\mathrm{time}\;\mathrm{of}\;\mathrm{stim}\mathrm{ulus}}{\begin{array}{c} \mathrm{expl}\mathrm{orat}\mathrm{ion}\;\mathrm{time}\;\mathrm{of}\;\mathrm{stim}\mathrm{ulus}\;1\\ \;+\mathrm{expl}\mathrm{orat}\mathrm{ion}\;\mathrm{time}\;\mathrm{of}\;\mathrm{stim}\mathrm{ulus}\;2 \end{array}}\right) \end{array}$$

### 4.7 Statistical Analysis

All statistical tests were performed using GraphPad Prism version 8.0.0 (GraphPad Software). Outlier tests were carried out beforehand. Shapiro wilk and Levene Tests were performed to test the normality and the equality of variance. For calcium imaging data, parametrical tests (unpaired & paired *t*‐test & One way ANOVA) were performed if data showed normal distribution and equality of group variances. Otherwise, non‐parametric tests (Mann–Whitney U test & Wilcoxon) were used. For behavior tests, statistical analyses were performed within sex to examine the effect of phenotype (OTR cKO). One way Anova or a non‐parametric equivalent were performed to compare between phenotypes, and one sample *t*‐test against chance (50%) to evaluate significant preferences in the conditioned place preference and sociability and social recognition tests. For one sample *t*‐test, p‐values are presented as ^*^ *p* < 0.05, ^**^ *p* < 0.01, ^***^ *p* < 0.001. For unpaired tests, p‐value are presented as ^#^ *p* < 0.05, ^##^ *p* < 0.01, ^###^ *p* < 0.001. All values, group compositions, and statistical tests for each experiment are detailed in Tables S1–S6.

## Author Contributions

CD: Ex vivo calcium imaging experiment and analysis, viral injections, behavioral assays analysis and experimental conception, writing of manuscript, figures composition, P‐AD: Ex vivo calcium imaging analysis, CM: behavioral assays analysis, SS: RNAscope. three‐dimensional reconstruction, slide scanner, data analysis, KW: calcium imaging, viral injection, AK: RNAscope, AB: calcium imaging, data analysis, YP: confocal imaging, slide scanner, MK, AC: behavioral assays, JH: immunohistochemistry, data analysis, ST: slide scanner, immunohistochemistry, AW: immunohistochemistry, confocal imaging, SW: RNAscope, FS: immunohistochemistry, confocal imaging, data analysis, MW: confocal imaging, TS: slide scanner, confocal imaging, writing of custom code, data analysis, FF: confocal imaging, slide scanner, data analysis, JM: RNAscope, confocal imaging, EE: RNAscope, slide scanner, CB: calcium imaging, data analysis, PD: supervision, QK: Confocal imaging, data analysis, CG: slide scanner, HF: supervision, data analysis, MKK: data analysis, VG: supervision, funding, PDb, JS: supervision, FA: project conception and administration, supervision, funding, writing of manuscript AC: project conception and administration, supervision, funding, writing of manuscript.

## Conflicts of Interest

The authors declare no conflicts of interest.

## Supporting information




**Supporting File 1**: advs75812‐sup‐0001‐SuppMat.pdf.

**Supporting File 2**: advs75812‐sup‐0002‐Data.xlsx.

**Supporting File 3**: advs75812‐sup‐0003‐TablesS1–S6.xlsx.

## Data Availability

The raw data generated in this study are available at public database under accession code (https://zenodo.org/records/20328439). Codes used for data analysis are available under accession code https://github.com/Team‐Charlet/Denis_et_al._2026. Statistical data are provided in the Statistic Tables 1‐6. In addition, all data that support the findings of this study are available from the corresponding authors upon request. Source data are provided with this paper.

## References

[1] G. Gimpl and F. Fahrenholz , “The Oxytocin Receptor System: Structure, Function, and Regulation,” Physiological Reviews 81, no. 2 (2001): 629–683, 10.1152/physrev.2001.81.2.629. 11274341

[2] H. J. Lee , A. H. Macbeth , J. H. Pagani , and W. S. Young , “Oxytocin: The Great Facilitator of Life,” Progress in Neurobiology 88, no. 2 (2009): 127, 10.1016/j.pneurobio.2009.04.001. 19482229 PMC2689929

[3] A. N. van den Pol , “Neuropeptide Transmission in Brain Circuits,” Neuron 76, no. 1 (2012): 98–115, 10.1016/j.neuron.2012.09.014. 23040809 PMC3918222

[4] D. Parmaksiz and Y. Kim , “Navigating Central Oxytocin Transport: Known Realms and Uncharted Territories,” The Neuroscientist 31 (2024): 234–261, 10.1177/10738584241268754. 39113465 PMC12103645

[5] M. Ludwig and G. Leng , “Dendritic Peptide Release and Peptide‐Dependent Behaviours,” Nature Reviews Neuroscience 7, no. 2 (2006): 126–136, 10.1038/nrn1845. 16429122

[6] H. S. Knobloch , A. Charlet , L. C. Hoffmann , et al., “Evoked Axonal Oxytocin Release in the Central Amygdala Attenuates Fear Response,” Neuron 73, no. 3 (2012): 553–566, 10.1016/j.neuron.2011.11.030. 22325206

[7] A. Verkhratsky and M. Nedergaard , “Physiology of Astroglia,” Physiological Reviews 98, no. 1 (2018): 239–389, 10.1152/physrev.00042.2016. 29351512 PMC6050349

[8] A. Araque , G. Carmignoto , P. G. Haydon , S. H. Oliet , R. Robitaille , and A. Volterra , “Gliotransmitters Travel in Time and Space,” Neuron 81, no. 4 (2014): 728–739, 10.1016/j.neuron.2014.02.007. 24559669 PMC4107238

[9] M. Santello , N. Toni , and A. Volterra , “Astrocyte Function From Information Processing to Cognition and Cognitive Impairment,” Nature Neuroscience 22, no. 2 (2019): 154–166, 10.1038/s41593-018-0325-8. 30664773

[10] N. Bazargani and D. Attwell , “Astrocyte Calcium Signaling: The Third Wave,” Nature Neuroscience 19, no. 2 (2016): 182–189, 10.1038/nn.4201. 26814587

[11] D. Di Scala‐Guenot and M. T. Strosser , “Oxytocin Receptors on Cultured Astroglial Cells. Kinetic and Pharmacological Characterization of Oxytocin‐Binding Sites on Intact Hypothalamic and Hippocampic Cells From Foetal Rat Brain,” Biochemical Journal 284 (1992): 491–497, 10.1042/bj2840491. 1318031 PMC1132665

[12] J. Wahis , A. Baudon , F. Althammer , et al., “Astrocytes Mediate the Effect of Oxytocin in the Central Amygdala on Neuronal Activity and Affective States in Rodents,” Nature Neuroscience 24 (2021): 529–541, 10.1038/s41593-021-00800-0. 33589833

[13] A. Baudon , V. Grelot , K. Y. Wang , et al., “Stress Induces Oxytocin‐Gαi‐Dependent Remodeling of Astrocytes to Shape Neuronal Response in the Amygdala,” Nature Communications 17, no. 1 (2025): 1364, 10.1038/s41467-025-68114-4. PMC12877189 41462022

[14] A. Baudon , E. Clauss Creusot , F. Althammer , C. P. Schaaf , and A. Charlet , “Emerging Role of Astrocytes in Oxytocin‐Mediated Control of Neural Circuits and Brain Functions,” Progress in Neurobiology 217 (2022): 102328, 10.1016/j.pneurobio.2022.102328. 35870680

[15] D. T. Theodosis , C. Montagnese , F. Rodriguez , J. D. Vincent , and D. A. Poulain , “Oxytocin Induces Morphological Plasticity in the Adult Hypothalamo‐Neurohypophysial System,” Nature 322, no. 6081 (1986): 738–740, 10.1038/322738a0. 3748154

[16] C. P. Meinung , L. Boi , S. Pandamooz , et al., “OXTR‐Mediated Signaling in Astrocytes Contributes to Anxiolysis,” Molecular Psychiatry 30 (2024): 2620–2634, 10.1038/s41380-024-02870-5. 39702695 PMC12092269

[17] D. Di Scala‐Guenot and M. T. Strosser , “Oxytocin Receptors on cultured astroglial cells. Regulation by a Guanine‐Nucleotide‐Binding Protein and Effect of Mg^2+^ ,” Biochemical Journal 284 (1992): 499–505, 10.1042/bj2840499. 1318032 PMC1132666

[18] F. Althammer , R. K. Roy , A. Lefevre , et al., “Altered PVN‐to‐CA2 Hippocampal Oxytocin Pathway and Reduced Number of Oxytocin‐Receptor Expressing Astrocytes in Heart Failure Rats,” Journal of Neuroendocrinology 34 (2022): 13166, 10.1111/jne.13166. PMC9495289 35657290

[19] H. E. Ross , S. M. Freeman , L. L. Spiegel , X. Ren , E. F. Terwilliger , and L. J. Young , “Variation in Oxytocin Receptor Density in the Nucleus Accumbens Has Differential Effects on Affiliative Behaviors in Monogamous and Polygamous Voles,” The Journal of Neuroscience 29, no. 5 (2009): 1312–1318, 10.1523/JNEUROSCI.5039-08.2009. 19193878 PMC2768419

[20] G. Dolen , A. Darvishzadeh , K. W. Huang , and R. C. Malenka , “Social Reward Requires Coordinated Activity of Nucleus Accumbens Oxytocin and Serotonin,” Nature 501, no. 7466 (2013): 179–184, 10.1038/nature12518. 24025838 PMC4091761

[21] D. Wei , D. Lee , C. D. Cox , et al., “Endocannabinoid Signaling Mediates Oxytocin‐Driven Social Reward,” Proceedings of the National Academy of Sciences 112, no. 45 (2015): 14084–14089, 10.1073/pnas.1509795112. PMC4653148 26504214

[22] M. Corkrum , A. Covelo , J. Lines , et al., “Dopamine‐Evoked Synaptic Regulation in the Nucleus Accumbens Requires Astrocyte Activity,” Neuron 105, no. 6 (2020): 1036–1047, 10.1016/j.neuron.2019.12.026. 31954621 PMC7322729

[23] C. Escartin , E. Galea , A. Lakatos , et al., “Reactive Astrocyte Nomenclature, Definitions, and Future Directions,” Nature Neuroscience 24, no. 3 (2021): 312–325, 10.1038/s41593-020-00783-4. 33589835 PMC8007081

[24] K. B. Casper and K. D. McCarthy , “GFAP‐Positive Progenitor Cells Produce Neurons and Oligodendrocytes Throughout the CNS,” Molecular and Cellular Neuroscience 31, no. 4 (2006): 676–684, 10.1016/j.mcn.2005.12.006. 16458536

[25] F. Althammer , E. G. Krause , A. D. de Kloet , et al., “Identification and Three‐Dimensional Reconstruction of Oxytocin Receptor Expressing Astrocytes in the Rat and Mouse Brain,” STAR Protocols 3, no. 1 (2022): 101160, 10.1016/j.xpro.2022.101160. 35199030 PMC8844904

[26] A. Koussounadis , S. P. Langdon , I. H. Um , D. J. Harrison , and V. A. Smith , “Relationship Between Differentially Expressed mRNA and mRNA‐Protein Correlations in a Xenograft Model System,” Scientific Reports 5 (2015): 10775, 10.1038/srep10775. 26053859 PMC4459080

[27] A. Baudon , E. Clauss‐Creusot , P. Darbon , R. Patwell , V. Grinevich , and A. Charlet , “Calcium Imaging and BAPTA Loading of Amygdala Astrocytes in Mouse Brain Slices,” STAR Protocols 3, no. 1 (2022): 101159, 10.1016/j.xpro.2022.101159. 35199029 PMC8844720

[28] S. S. Moy , J. J. Nadler , A. Perez , et al., “Sociability and Preference for Social Novelty in Five Inbred Strains: An Approach to Assess Autistic‐Like Behavior in Mice,” Genes, Brain and Behavior 3, no. 5 (2004): 287–302, 10.1111/j.1601-1848.2004.00076.x. 15344922

[29] A. M. Reeves , E. Shigetomi , and B. S. Khakh , “Bulk Loading of Calcium Indicator Dyes to Study Astrocyte Physiology: Key Limitations and Improvements Using Morphological Maps,” Journal of Neuroscience 31, no. 25 (2011): 9353–9358, 10.1523/JNEUROSCI.0127-11.2011. 21697385 PMC3142876

[30] F. Althammer , R. K. Roy , M. K. Kirchner , et al., “Impaired Oxytocin Signalling in the Central Amygdala in Rats With Chronic Heart Failure,” The Journal of Physiology 602, no. 22 (2024): 6259–6280, 10.1113/JP286297. 39530490 PMC11576253

[31] F. Althammer , M. C. Wimmer , Q. Krabichler , et al., “Analysis of the Hypothalamic Oxytocin System and Oxytocin Receptor‐Expressing Astrocytes in a Mouse Model of Prader–Willi Syndrome,” Journal of Neuroendocrinology 34, no. 12 (2022): 13217, 10.1111/jne.13217. 36458331

[32] P. Wang , D. Qin , and Y. F. Wang , “Oxytocin Rapidly Changes Astrocytic GFAP Plasticity by Differentially Modulating the Expressions of pERK 1/2 and Protein Kinase A,” Frontiers in Molecular Neuroscience 10 (2017): 262, 10.3389/fnmol.2017.00262. 28860967 PMC5559427

[33] A. Baudon , V. Grelot , K. Y. Wang , et al., “Stress Induces Oxytocin‐Gαi‐Dependent Remodeling of Astrocytes to Shape Neuronal Response in the Amygdala,” Nature Communications 17 (2025): 1364, 10.1038/s41467-025-68114-4. PMC12877189 41462022

[34] N. Mazare , M. Oudart , J. Moulard , et al., “Local Translation in Perisynaptic Astrocytic Processes Is Specific and Changes After Fear Conditioning,” Cell Reports 32, no. 8 (2020): 108076, 10.1016/j.celrep.2020.108076. 32846133 PMC7450274

[35] K. Sakers , A. M. Lake , R. Khazanchi , et al., “Astrocytes Locally Translate Transcripts in Their Peripheral Processes,” Proceedings of the National Academy of Sciences 114, no. 19 (2017): E3830, 10.1073/pnas.1617782114. PMC5441704 28439016

[36] H. E. Ross , C. D. Cole , Y. Smith , et al., “Characterization of the Oxytocin System Regulating Affiliative Behavior in Female Prairie Voles,” Neuroscience 162, no. 4 (2009): 892–903, 10.1016/j.neuroscience.2009.05.055. 19482070 PMC2744157

[37] H. Arakawa and M. Tokashiki , “The Posterior Intralaminar Thalamic Nucleus Promotes Nose‐to‐Nose Contacts Leading to Prosocial Reception in the Sequence of Mouse Social Interaction,” European Journal of Neuroscience 60, no. 7 (2024): 5731–5749, 10.1111/ejn.16520. 39210622

[38] H. Arakawa , D. C. Blanchard , and R. J. Blanchard , “Colony Formation of C57BL/6J Mice in Visible Burrow System: Identification of Eusocial Behaviors in a Background Strain for Genetic Animal Models of Autism,” Behavioural Brain Research 176, no. 1 (2007): 27–39, 10.1016/j.bbr.2006.07.027. 16971001 PMC3264663

[39] H. Arakawa , S. Cruz , and T. Deak , “From Models to Mechanisms: Odorant Communication as a Key Determinant of Social Behavior in Rodents During Illness‐Associated States,” Neuroscience & Biobehavioral Reviews 35, no. 9 (2011): 1916–1928, 10.1016/j.neubiorev.2011.03.007. 21414355

[40] T. L. Bale and D. M. Dorsa , “Sex Differences in and Effects of Estrogen on Oxytocin Receptor Messenger Ribonucleic Acid Expression in the Ventromedial Hypothalamus,” Endocrinology 136, no. 1 (1995): 27–32, 10.1210/endo.136.1.7828541. 7828541

[41] A. E. Johnson , H. Coirini , T. R. Insel , and B. S. McEwen , “The Regulation of Oxytocin Receptor Binding in the Ventromedial Hypothalaimic Nucleus by Testosterone and Its Metabolites*,” Endocrinology 128, no. 2 (1991): 891–896, 10.1210/endo-128-2-891. 1846593

[42] A. B. Chen , M. Duque , A. Rymbek , et al., “Norepinephrine Changes Behavioral State Through Astroglial Purinergic Signaling,” Science 388, no. 6748 (2025): 769–775, 10.1126/science.adq5233. 40373133 PMC12265949

[43] K. A. Guttenplan , I. Maxwell , E. Santos , et al., “GPCR Signaling Gates Astrocyte Responsiveness to Neurotransmitters and Control of Neuronal Activity,” Science 388, no. 6748 (2025): 763–768, 10.1126/science.adq5729. 40373148 PMC13132101

[44] K. B. Lefton , Y. Wu , Y. Dai , et al., “Norepinephrine Signals Through Astrocytes to Modulate Synapses,” Science 388, no. 6748 (2025): 776–783, 10.1126/science.adq5480. 40373122 PMC12309572

[45] L. W. Hung , S. Neuner , J. S. Polepalli , et al., “Gating of Social Reward by Oxytocin in the Ventral Tegmental Area,” Science 357, no. 6358 (2017): 1406–1411, 10.1126/science.aan4994. 28963257 PMC6214365

[46] S. Valtcheva , H. A. Issa , C. J. Bair‐Marshall , et al., “Neural Circuitry for Maternal Oxytocin Release Induced by Infant Cries,” Nature 621, no. 7980 (2023): 788–795, 10.1038/s41586-023-06540-4. 37730989 PMC10639004

[47] I. Carcea , N. L. Caraballo , B. J. Marlin , et al., “Oxytocin Neurons Enable Social Transmission of Maternal Behaviour,” Nature 596, no. 7873 (2021): 553–557, 10.1038/s41586-021-03814-7. 34381215 PMC8387235

[48] B. J. Marlin , M. Mitre , A. D'Amour J , M. V. Chao , and R. C. Froemke , “Oxytocin Enables Maternal Behaviour by Balancing Cortical Inhibition,” Nature 520, no. 7548 (2015): 499, 10.1038/nature14402. 25874674 PMC4409554

[49] C. L. Ford and L. J. Young , “Refining Oxytocin Therapy for Autism: Context is Key,” Nature Reviews Neurology 18 (2021): 67–68, 10.1038/s41582-021-00602-9. PMC8816821 34880473

[50] C. Edmonson , M. N. Ziats , and O. M. Rennert , “Altered Glial Marker Expression in Autistic Post‐Mortem Prefrontal Cortex and Cerebellum,” Molecular Autism 5, no. 1 (2014): 3, 10.1186/2040-2392-5-3. 24410870 PMC3914711

[51] M. Allen , B. S. Huang , M. J. Notaras , et al., “Astrocytes Derived From ASD Individuals Alter Behavior and Destabilize Neuronal Activity Through Aberrant Ca^2+^ Signaling,” Molecular Psychiatry 27, no. 5 (2022): 2470–2484, 10.1038/s41380-022-01486-x. 35365802 PMC9135629

