# Supplementary material for: A Brain‐Wide Atlas of Astrocytic Oxytocin Receptors Reveals a Glial Basis for Nucleus Accumbens Modulation of Affiliative Behavior
Source: Adv Sci (Weinh). 2026 Jun 4:e18450. Online ahead of print. doi: 10.1002/advs.202518450 (PMC13336011; doi:10.1002/advs.202518450)
Supplement: Supplementary file 1 — Supporting File 1: advs75812‐sup‐0001‐SuppMat.pdf. [file ADVS-9999-e18450-s002.pdf]

A Brain-Wide Atlas of Astrocytic Oxytocin Receptors in Mouse and Rat Reveals a Glial Basis for Nucleus Accumbens Modulation of Affiliative Behavior

Clémence Denis[1*], Stefan Stojilkovic[2*], Kai-Yi Wang[1], Cristina Márquez[3,4], Annabel C. Kleinwächter[2], Angel Baudon[1], Yuval Podpecan[2], Aurélia Ces[1], Mélanie Kremer[1], Isabelle Arnoux[5], Nathalie Rouach[5] Jemima Helen[2], Sophie Trender[2], Andreas Wallkum[2], Selina Wunsch[2], Franziska Schommer[2], Moritz C. Wimmer[2], Tim Schubert[2], Felix Franke[2], Jabir Aliyu Muhammad[2], Eva M. Eisemann[2], Ingrid Camila Possa-Paranhos[2], Cassandra Baumann[1], Pierre-Alexis Derrien[1], Quirin Krabichler[6], Cosmo Garcia[7], Henning Fröhlich[2], Matthew K. Kirchner[7], Valery Grinevich[6], Pascal Darbon[1], Javier E. Stern[7], Ferdinand Althammer[2§#], Alexandre Charlet[1§#]

Affiliations:

[1] Centre National de la Recherche Scientifique and University of Strasbourg, Institute of Cellular and Integrative Neuroscience, 67000 Strasbourg, France

[2] Institute of Human Genetics, Heidelberg University, 69120 Heidelberg, Germany

[3] Center for Neuroscience and Cell Biology CNC-UC, University of Coimbra, Portugal

[4] Centre for Innovative Biomedicine and Biotechnology, University of Coimbra, Portugal

[5] Center for Interdisciplinary Research in Biology, Collège de France, CNRS, INSERM, PSL-Neuro, Université PSL, Paris, France.

[6] Department of Neuropeptide Research in Psychiatry, Central Institute of Mental Health, German Center for Psychiatry, Medical Faculty Mannheim, University of Heidelberg, Mannheim 68159, Germany

[7] Center for Neuroinflammation and Cardiometabolic Diseases, and Neuroscience Institute, Georgia State University, Atlanta, GA, USA

*These authors contributed equally

§These authors jointly supervised the work

#Correspondence acharlet@unistra.fr; ferdinand.althammer@med.uni-heidelberg.de

Supplementary Figures and figures legends

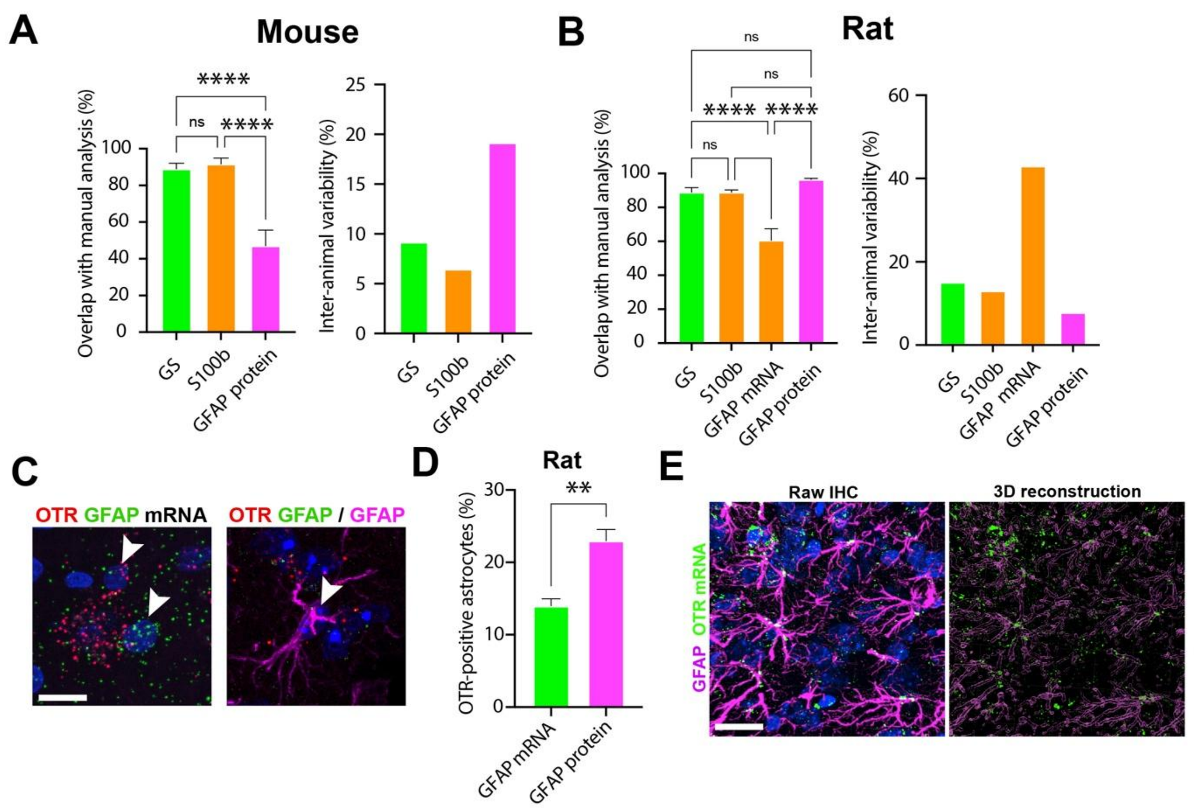

Figure S1:

A Functional validation of our automated pipeline to assess and quantify OTR+ astrocytes across various brain regions in the mouse. Left graph shows the overlap with manual analysis, left graph depicts inter-animal variability based on different astrocyte-specific antibodies. **B** Functional validation of our automated pipeline to assess and quantify OTR+ astrocytes across various brain regions in the rat. Left graph shows the overlap with manual analysis, left graph depicts inter-animal variability based on different astrocyte-specific antibodies. **C** Depiction of different approaches to assess OTR+ astrocytes in the mouse and rat brain. Left confocal images shows multiplexed RNAscope using OTR and GFAP mRNA probes, right image shows a combinatory approach of multiplexed RNAscope using OTR and GFAP mRNA probes combined with GFAP protein antibody staining. **D** Quantification of OTR+ astrocytes using GFAP mRNA or GFAP protein as the sole astrocyte marker. **E** Overview images shows distribution of OTR+ astrocytes in the rat CeA. Left images shows the raw confocal image, left images highlights three-dimensional reconstructions of the same astrocytes.

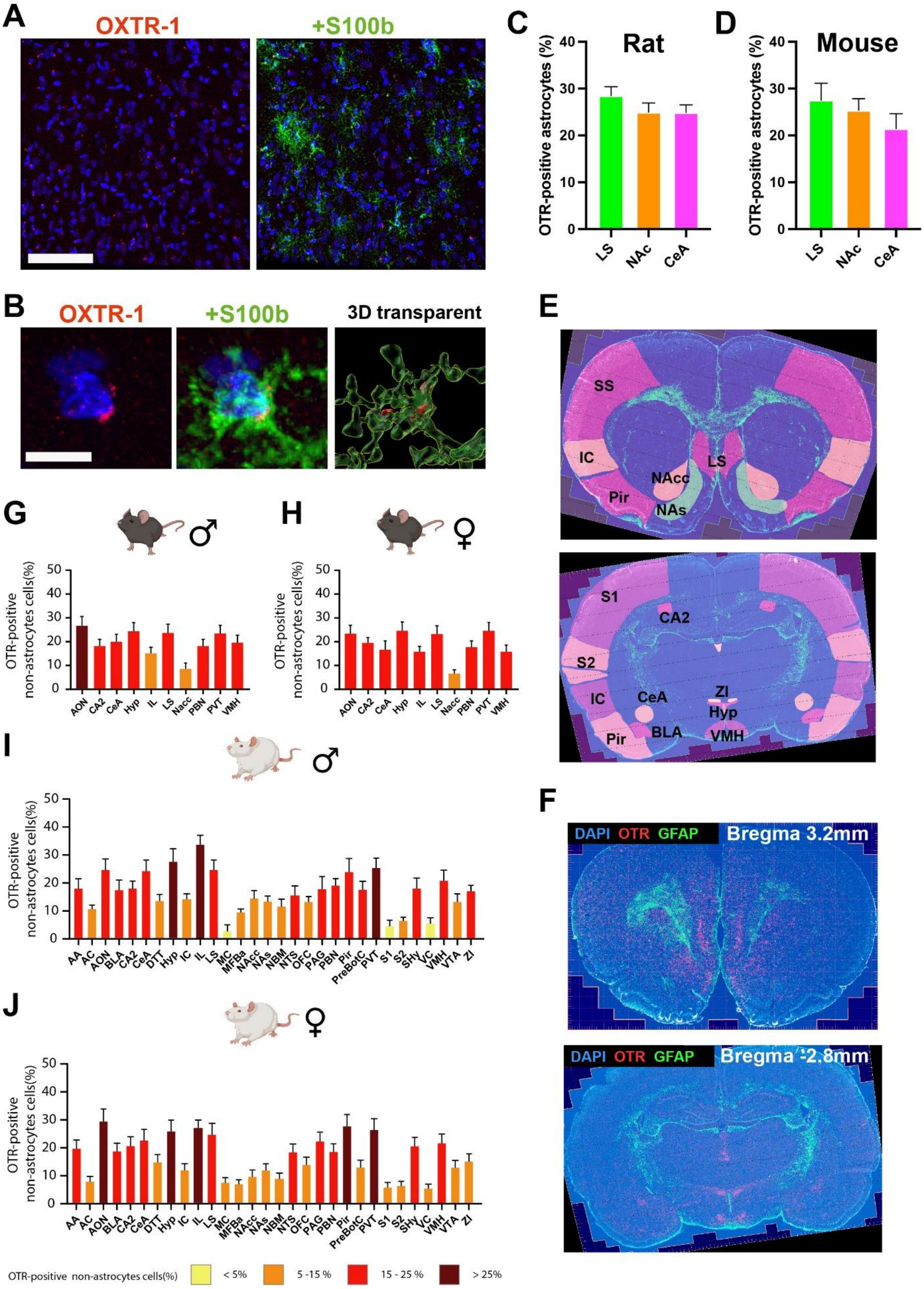
A
OXTR-1
+S100b
B
OXTR-1
+S100b
3D transparent
C
Rat
OTR-positive astrocytes (%)
LS
NAc
CeA
D
Mouse
OTR-positive astrocytes (%)
LS
NAc
CeA
E
SS
IC
LS
NAcc
Pir
NAs
S1
CA2
S2
IC
CeA
ZI
Hyp
Pir
BLA
VMH
F
DAPI OTR GFAP Bregma 3.2mm
DAPI OTR GFAP Bregma -2.8mm
G
H
OTR-positive non-astrocytes cells(%)
AON CA2 CeA Hyp IL LS Nacc PBN PVT VMH
I
J
OTR-positive non-astrocytes cells(%)
AA AC AON BLA CA2 CeA DTT Hyp IC IL LS MC MFBa NAcc NAs NBM NTS OFC PAG PBN Pir PreBotC PVT S1 S2 SHy VC VMH VTA ZI
OTR-positive non-astrocytes cells(%)
< 5%
5 -15 %
15 - 25 %
> 25%

<u>Figure S2:</u> S2 Validation of astrocytic OTR expression via OTR-specific antibody and Methodological approach for OTR+ astrocytes detection

A. Confocal overview images show the distribution of OTR-positive cells within the CeA of a rat. Scale bar = 200µm. **B.** High magnification images show an OTR-positive astrocyte labeled via the astrocyte marker S100b. The transparent three-dimensional reconstruction shows that OTR expression is confined to the astrocyte body. Scale bar = 10µm. **C.** Quantification of OTR+ astrocytes in various brain regions of the rat. **D.** Quantification of OTR+ astrocytes in various brain regions of the mouse. **E.** Overview of rat coronal brain sections highlighting all brain regions analyzed for OTR+ astrocytes. **F.** Overview of rat coronal brain sections showing GFAP and OTR mRNA signal distribution across the entire brain slide. **G.** OTR+ non-astrocytic cells in different brain regions in male mice. **H.** OTR+ non-astrocytic cells in different brain regions in female mice. **I.** OTR+ non-astrocytic cells in different brain regions in male rats. **J.** OTR+ non-astrocytic cells in different brain regions in female rats. BLA-Basolateral Amygdala; CA2-Ammon's horn 2; CeA-Central Amygdala; IC-Insular Cortex; Hyp-Hypothalamus (mostly paraventricular nucleus9; Pir-Piriform cortex; SS-Somatosensory cortex; VMH-Vendromedian Hypothalamus; ZI-Zona Incerta

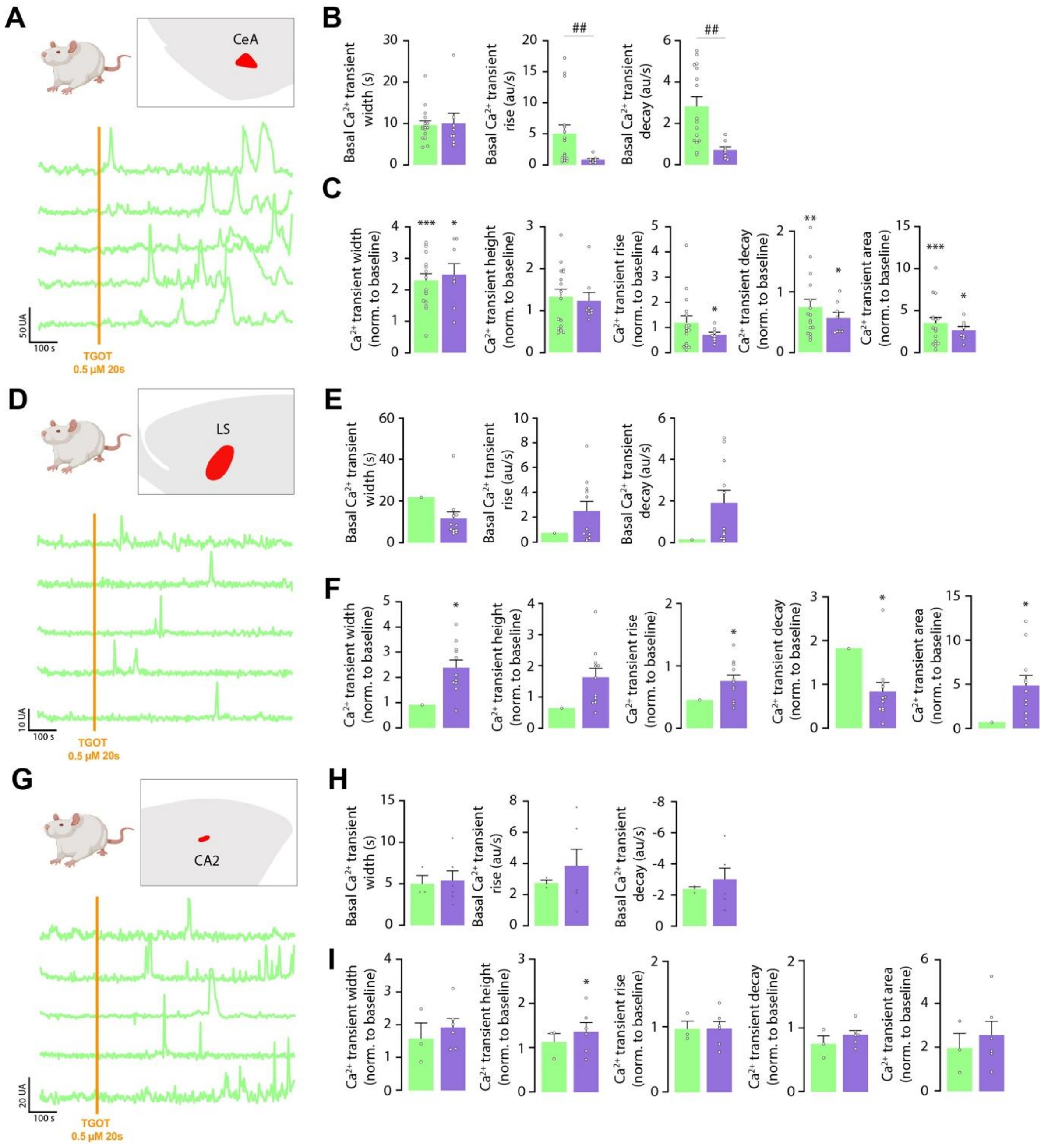

<u>Figure S3:</u> Anatomical mapping and functional assessment of OTR+ astrocytes in rat CeA, LS and CA2

A. Typical ΔF traces of OGB1 fluorescence intensity of individual astrocytes in response to TGOT (500 nM) + TTX (1µM) in males. The vertical line indicates 20s of TGOT application. **B**. Bar plots showing mean calcium transient width, rise and decay in male and females during baseline time ($n_{\text{astro resp male CeA}}$=54, $n_{\text{astro resp female CeA}}$=47, $n_{\text{rat resp CeA}}$ =3-8). **C**. Bar plots showing calcium transient height, width, rise, decay and area triggered by TGOT application in male (green) and females (purple) normalized to baseline values ($n_{\text{astro resp male CeA}}$=54, $n_{\text{astro resp female CeA}}$=47, $n_{\text{rat resp CeA}}$ =3-8). **D**. Typical ΔF traces of OGB1 fluorescence intensity of individual astrocytes in response to TGOT (500 nM) + TTX (1µM) in males. The vertical line indicates

20s of TGOT application. **E**. Bar plots showing mean calcium transient width, rise and decay in male and females during baseline time ($n_{astro\ resp\ male\ LS}$=14, $n_{astro\ resp\ female\ LS}$=24, $n_{rat\ resp\ LS}$ =3-5). **F**. Bar plots showing calcium transient height, width, rise, decay and area triggered by TGOT application in male and females normalized to baseline values ($n_{astro\ resp\ male\ LS}$=14, $n_{astro\ resp\ female\ LS}$=24, $n_{rat\ resp\ LS}$ =3-5). **G**. Typical ΔF traces of OGB1 fluorescence intensity of individual astrocytes in response to TGOT (500 nM) + TTX (1µM) in males. The vertical line indicates 20s of TGOT application. **H**. Bar plots showing mean calcium transient width, rise and decay in male and females during baseline time ($n_{astro\ resp\ male\ CA2}$=26, $n_{astro\ resp\ female\ CA2}$=40, $n_{rat\ resp\ CA2}$ =3-5). **I**. Bar plots showing calcium transient height, width, rise, decay and area triggered by TGOT application in male and females normalized to baseline values ($n_{astro\ resp\ male\ CA2}$=26, $n_{astro\ resp\ female\ CA2}$=40, $n_{rat\ resp\ CA2}$ =3-5). Data are expressed as mean across animal ± SEM. Detailed statistics can be found in *Statistic Table 3*.. # p<0.05, ## p<0.01, ### p<0,001 & * p<0.05, ** p<0.01, *** p<0,001, two-sided unpaired *t*-test or Mann–Whitney U test or paired t-test or Wilcoxon test.

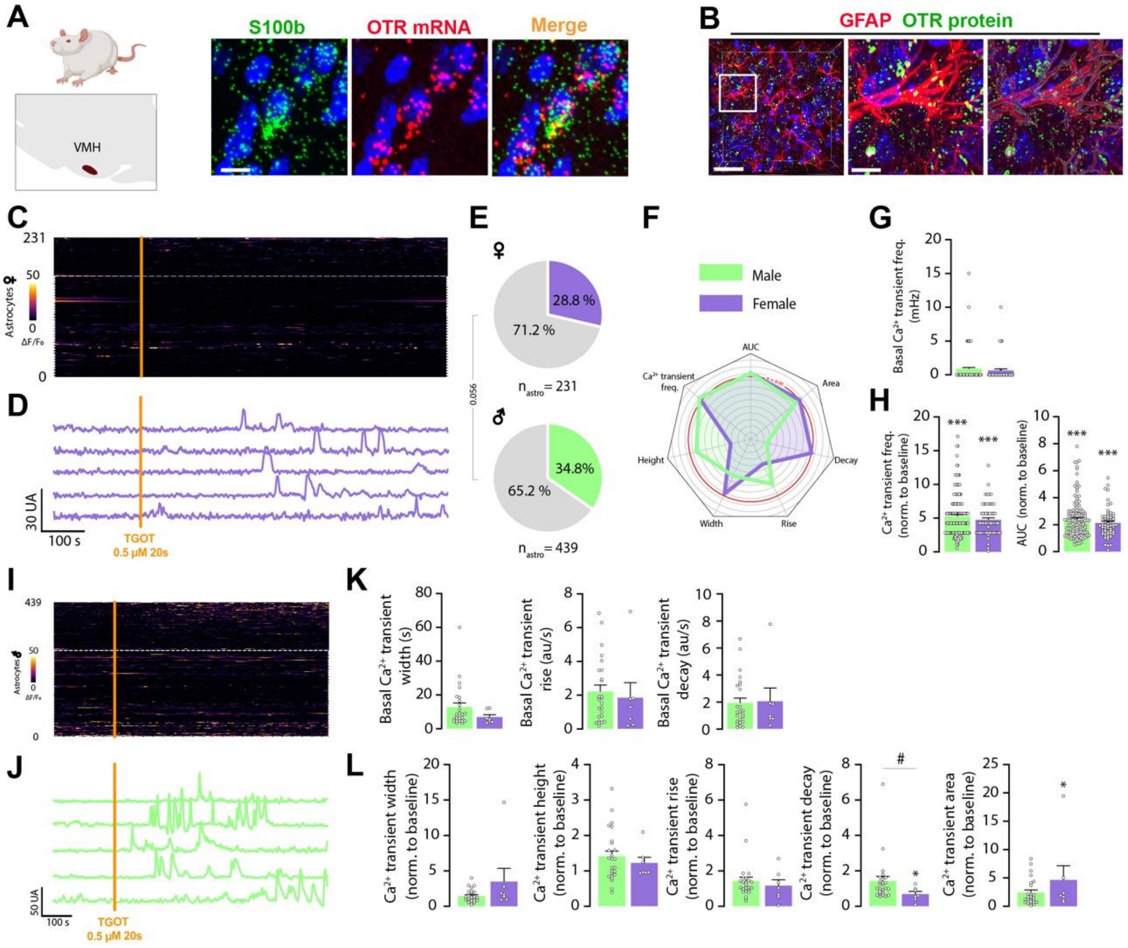

<u>Figure S4</u>: Detailed mapping of the astrocytic OTR in rat VMH

A. Schematic depiction of anatomical location of the rat VMH. Confocal images show a typical OTR+ astrocyte within the VMH. Scale bar = 10µm. **B**. Overview and high magnification of GFAP-stained, OTR+ astrocyte in the rat VMH. Scale bars = 100µm and 10µm. **C**. Heatmap of OGB1 fluorescence intensity of individual astrocytes in response to TGOT (500nM) in female. The vertical line indicates 20s of TGOT application. **D**. Typical ΔF traces of OGB1 fluorescence intensity of individual astrocytes in response to TGOT (500 nM) + TTX (1µM) in female. The vertical line indicates 20s of TGOT application. **E**. Proportion of VMH astrocytes responding to TGOT application in male and female individuals ($n_{astro\ male\ VMH}$=439, $n_{astro\ female\ VMH}$=231, $n_{rat\ VMH}$ =3-3). **F**. Comparison of calcium activity triggered by TGOT application in male and female individuals. Spider plots showing the p-value of the comparison between the basal state and after the application of TGOT for different calcium signaling parameters. **G**. Bar plot showing mean frequency of calcium transients in male and females during baseline time ($n_{astro\ resp\ male\ VMH}$=157$n_{astro\ resp\ female\ VMH}$=63, $n_{rat\ resp\ VMH}$ =3-3). **H**. Bar plots showing mean AUC and mean frequency of calcium transients triggered by TGOT application in male and females normalized to baseline values ($n_{astro\ resp\ male\ VMH}$=157, $n_{astro\ resp\ female\ VMH}$=63, $n_{rat\ resp\ VMH}$ =3-3). **I**. Heatmap of OGB1 fluorescence intensity of individual astrocytes in response to TGOT

(500nM) in male. The vertical line indicates 20s of TGOT application. **J**. Typical ΔF traces of OGB1 fluorescence intensity of individual astrocytes in response to TGOT (500 nM) + TTX (1μM) in males. The vertical line indicates 20s of TGOT application.**K**. Bar plots showing mean calcium transient width, rise, and decay in male and females during baseline time ($n_{astro\ resp\ male\ VMH}$=157, $n_{astro\ resp\ female\ VMH}$=63, $n_{rat\ resp\ VMH}$ =3-3). **L**. Bar plots showing calcium transient height, width, rise, decay and area triggered by TGOT application in male and females normalized to baseline values ($n_{astro\ resp\ male\ VMH}$=157, $n_{astro\ resp\ female\ VMH}$=63, $n_{rat\ resp\ VMH}$ =3-3). Data are expressed as mean across animal ± SEM. Detailed statistics can be found in *Statistic Table 3*. # p<0.05, ## p<0.01, ### p<0,001 & * p<0.05, ** p<0.01, *** p<0,001, two-sided unpaired *t*-test or Mann–Whitney U test or paired t-test or Wilcoxon test.

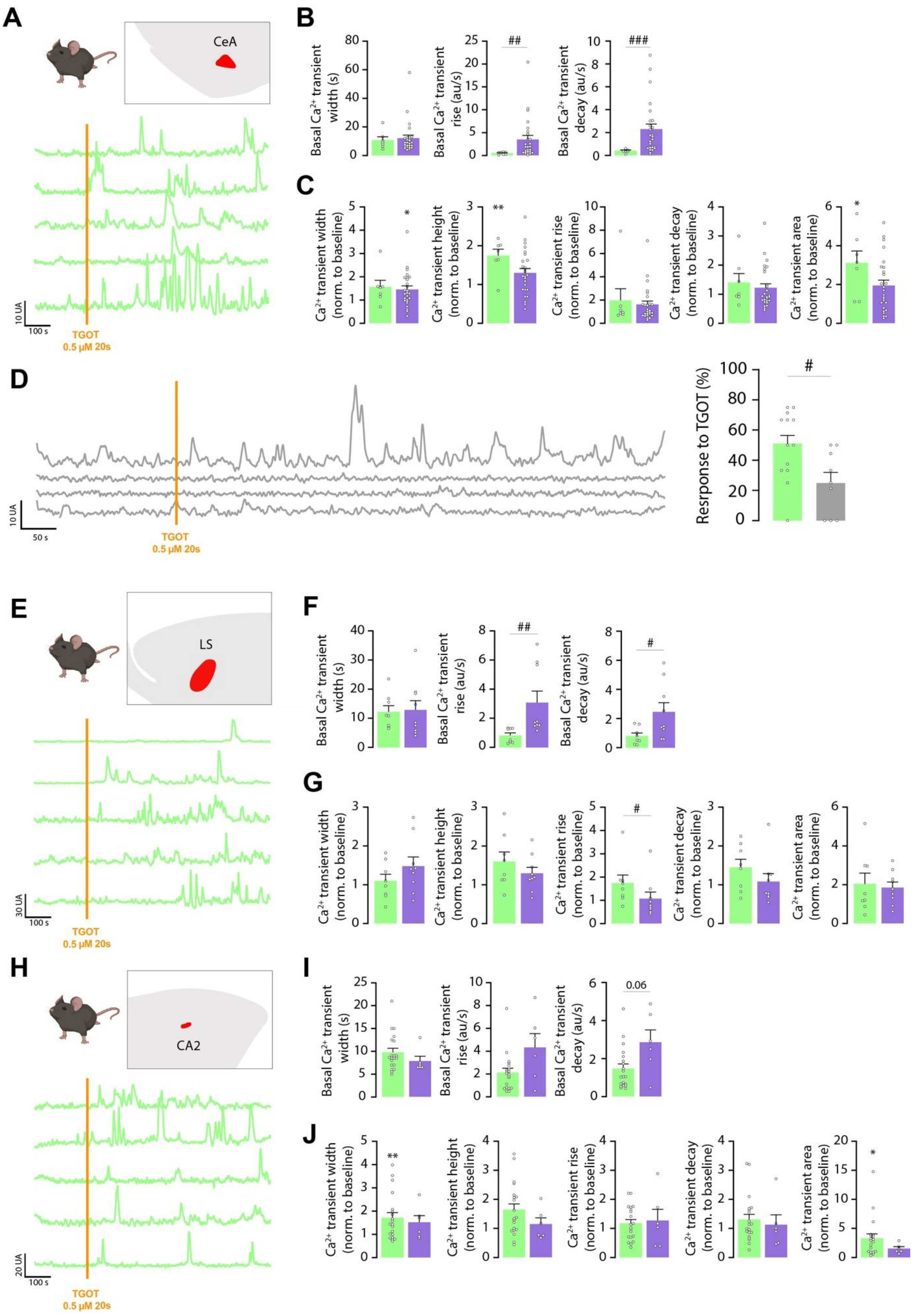
A
CeA
10 UA
100 s
TGOT
0.5 µM 20s
B
Basal Ca2+ transient width (s)
Basal Ca2+ transient rise (au/s)
Basal Ca2+ transient decay (au/s)
##
###
C
Ca2+ transient width (norm. to baseline)
Ca2+ transient height (norm. to baseline)
Ca2+ transient rise (norm. to baseline)
Ca2+ transient decay (norm. to baseline)
Ca2+ transient area (norm. to baseline)
D
10 UA
50 s
TGOT
0.5 µM 20s
Resrponse to TGOT (%)
#
E
LS
30 UA
100 s
TGOT
0.5 µM 20s
F
Basal Ca2+ transient width (s)
Basal Ca2+ transient rise (au/s)
Basal Ca2+ transient decay (au/s)
##
#
G
Ca2+ transient width (norm. to baseline)
Ca2+ transient height (norm. to baseline)
Ca2+ transient rise (norm. to baseline)
Ca2+ transient decay (norm. to baseline)
Ca2+ transient area (norm. to baseline)
#
H
CA2
20 UA
100 s
TGOT
0.5 µM 20s
I
Basal Ca2+ transient width (s)
Basal Ca2+ transient rise (au/s)
Basal Ca2+ transient decay (au/s)
0.06
J
Ca2+ transient width (norm. to baseline)
Ca2+ transient height (norm. to baseline)
Ca2+ transient rise (norm. to baseline)
Ca2+ transient decay (norm. to baseline)
Ca2+ transient area (norm. to baseline)

Figure S5: Anatomical mapping and functional assessment of OTR+ astrocytes in mice CeA, LS and CA2

A. Typical ΔF traces of OGB1 fluorescence intensity of individual astrocytes in response to TGOT (500 nM) + TTX (1μM) in males. The vertical line indicates 20s of TGOT application. **B**. Bar plots showing mean calcium transient width, rise and decay in male (green) and female (purple) during baseline time ($n_{astro\ resp\ male\ CeA}$=27, $n_{astro\ resp\ female\ CeA}$=78, $n_{mouse\ resp\ CeA}$ =3-3). **C**. Bar plots showing calcium transient height, width, rise, decay and area triggered by TGOT application in male and females normalized to baseline values ($n_{astro\ resp\ male\ CeA}$=27 $n_{astro\ resp\ female\ CeA}$=78, $n_{mouse\ resp\ CeA}$ =3-3). **D**. Typical ΔF traces of OGB1 fluorescence intensity of individual astrocytes in response to TGOT (500 nM) + TTX (1μM) + dOVT (1 μM) in males. The vertical line indicates 20s of TGOT application. Additionally, bar plot with proportion of CeA astrocytes responding to TGOT application in male without (green) or with (grey) OT antagonist in bath recording ($n_{astro\ male\ CTRL}$=54, $n_{astro\ male\ dOVT}$=48, $n_{rat\ CeA}$ =3-5). **E**. Typical ΔF traces of OGB1 fluorescence intensity of individual astrocytes in response to TGOT (500 nM) + TTX (1μM) in males. The vertical line indicates 20s of TGOT application. **F**. Bar plots showing mean calcium transient width, rise and decay in male and females during baseline time ($n_{astro\ resp\ male\ LS}$=27, $n_{astro\ resp\ female\ LS}$=22, $n_{mouse\ resp\ LS}$ =4-4). **G**. Bar plots showing calcium transient height, width, rise, decay and area triggered by TGOT application in male and females normalized to baseline values ($n_{astro\ resp\ male\ LS}$=27, $n_{astro\ resp\ female\ LS}$=22, $n_{mouse\ resp\ LS}$ =4-4). **H**. Typical ΔF traces of OGB1 fluorescence intensity of individual astrocytes in response to TGOT (500 nM) + TTX (1μM) in males. The vertical line indicates 20s of TGOT application. **I**. Bar plots showing mean calcium transient width, rise and decay in male and females during baseline time ($n_{astro\ resp\ male\ CA2}$=41, $n_{astro\ resp\ female\ CA2}$=28, $n_{mouse\ resp\ CA2}$ =3-4). **J**. Bar plots showing calcium transient height, width, rise, decay and area triggered by TGOT application in male and females normalized to baseline values ($n_{astro\ resp\ male\ CA2}$=41, $n_{astro\ resp\ female\ CA2}$=28, $n_{mouse\ resp\ CA2}$ =3-4). Data are expressed as mean across animal ± SEM. Detailed statistics can be found in *Statistic Table 4*. # p<0.05, ## p<0.01, ### p<0,001 & * p<0.05, ** p<0.01, *** p<0,001, two-sided unpaired *t*-test or Mann–Whitney U test or paired t-test or Wilcoxon test.

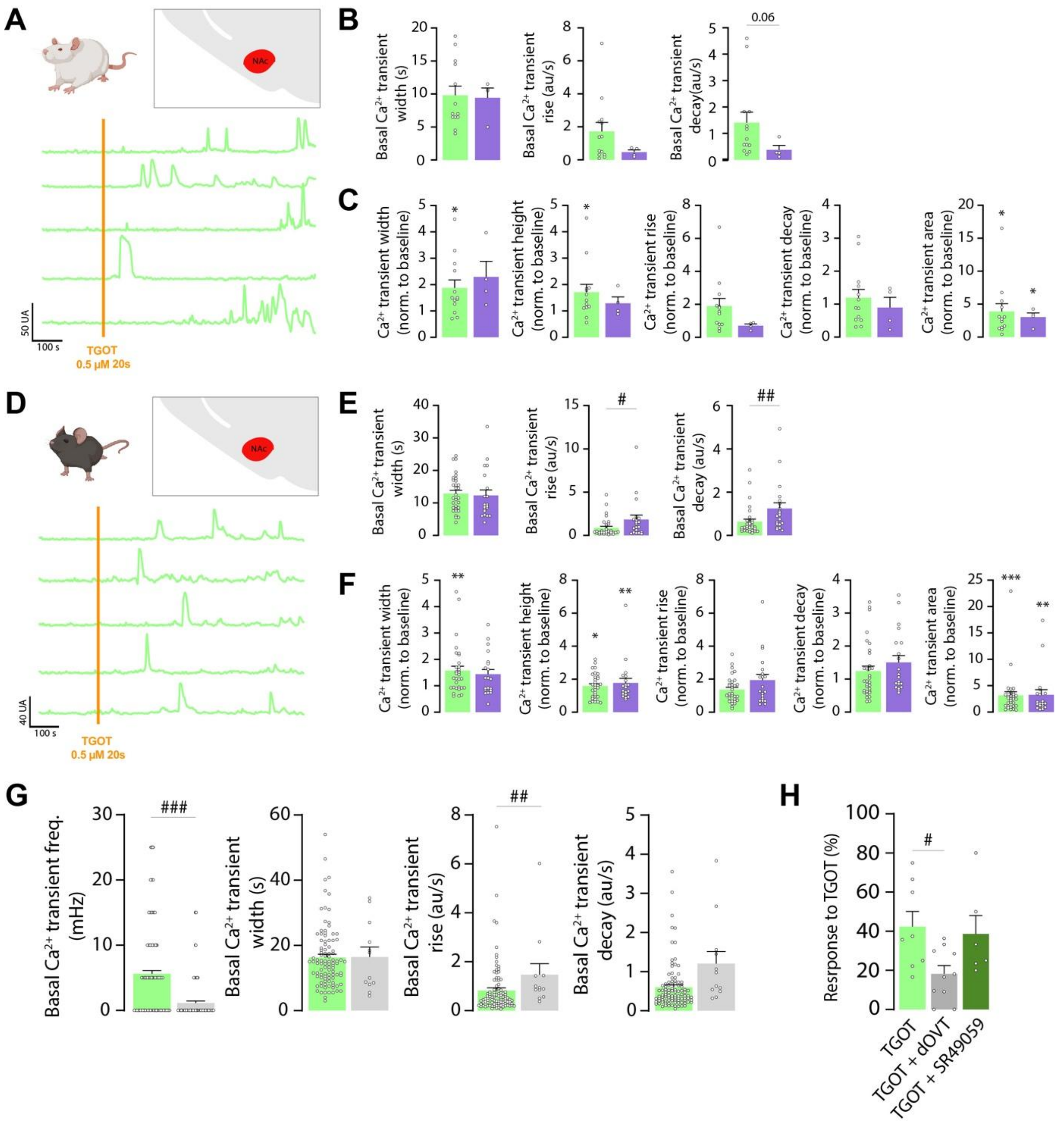

<u>Figure S6</u>: Detailed mapping of the astrocytic OTR in mice NAc

A. Typical ΔF traces of OGB1 fluorescence intensity of individual astrocytes in response to TGOT (500 nM) + TTX (1µM) in males. The vertical line indicates 20s of TGOT application. **B**. Bar plots showing mean calcium transient height, width, rise, decay and area in male and females during baseline time ($n_{\text{astro resp male NAc}}$ =29, $n_{\text{record resp male NAc}}$ = 27, $n_{\text{astro resp female NAc}}$ =23, $n_{\text{record resp female NAc}}$ = 16, $n_{\text{rat resp NAc}}$ =3-5). **C**. Bar plots showing calcium transient width, rise and decay triggered by TGOT application in male and females normalized to baseline values ($n_{\text{astro resp male NAc}}$ =29, $n_{\text{record resp male NAc}}$ = 27, $n_{\text{astro resp female NAc}}$ =23, $n_{\text{record resp female NAc}}$ = 16, $n_{\text{rat resp NAc}}$ =3-5). **D**. Typical ΔF traces of OGB1 fluorescence intensity of individual

astrocytes in response to TGOT (500 nM) + TTX (1µM) in males. The vertical line indicates 20s of TGOT application. **E**. Bar plots showing mean calcium transient width, rise and decay in male (green) and females (purple) during baseline time ($n_{astro\ resp\ male\ NAc}$ =124, $n_{record\ resp\ male\ NAc}$ = 22, $n_{astro\ resp\ female\ NAc}$ =87, $n_{record\ resp\ female\ NAc}$ = 16, $n_{mouse\ resp\ NAc}$ =5-3). **F**. Bar plots showing calcium transient height, width, rise, decay and area triggered by TGOT application in male and females normalized to baseline values ($n_{astro\ resp\ male\ NAc}$ =124, $n_{record\ resp\ male\ NAc}$ = 22, $n_{astro\ resp\ female\ NAc}$ =87, $n_{record\ resp\ female\ NAc}$ = 16, $n_{mouse\ resp\ NAc}$ =5-3). **G**. Bar plots showing calcium transient frequency, width, rise and decay triggered by TGOT application in control male and cKO male normalized to baseline values ($n_{astro\ CTRL\ male}$=179, $n_{astro\ cKO\ male}$=118, $n_{mouse\ NAc}$ =5-3). **H**. Bar plot with proportion of NAc astrocytes responding to TGOT puff application in male without (light green) or with (grey) OT antagonist or with (dark green) vasopressin antagonist (SR49059) in bath recording ($n_{astro\ male\ CTRL\ puff}$ =65, $n_{astro\ male\ dOVT\ puff}$ = 103, $n_{astro\ male\ SR49059\ puff}$ = 53, $n_{mouse\ NAc}$ =3-4-2) Data are expressed as mean across animal ± SEM. Detailed statistics can be found in *Statistic Table 5*. # p<0.05, ## p<0.01, ### p<0,001 & * p<0.05, ** p<0.01, *** p<0,001, two-sided unpaired *t*-test or Mann–Whitney U test or paired t-test or Wilcoxon test or anova one way with sidak's or tukey's post hoc test..

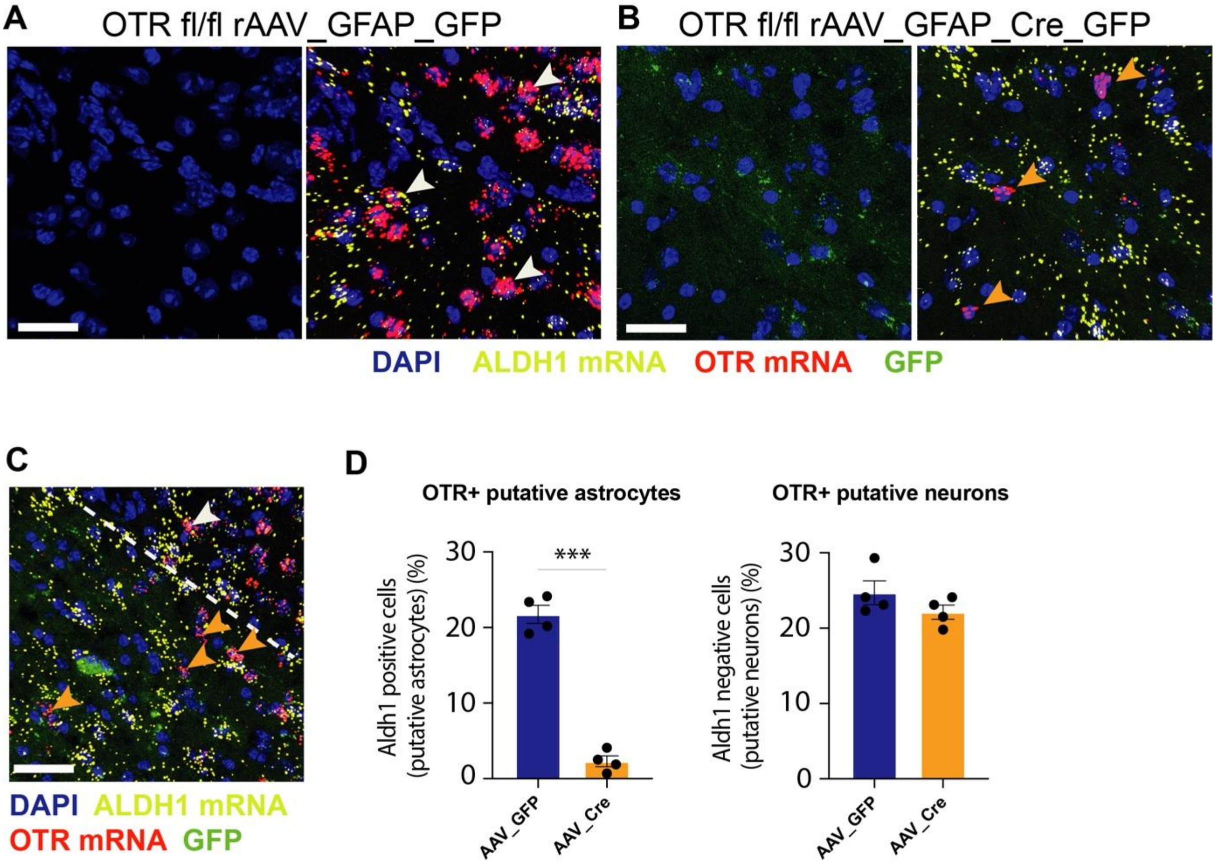

<u>Figure S7</u>: Validation of viral strategy for astrocytic OTR deletion in the NAc

A. Confocal images show multiplexed RNAscope for ALDH1 (yellow) and OTR (red) mRNA in the NAc of a non-injected control mouse. White arrowheads highlight OTR+ astrocytes. Scale bar = 25µm. **B.** Confocal images show multiplexed RNAscope for ALDH1 (yellow) and OTR (red) mRNA in the NAc of a mouse injected with AAV_GFAP_GFP_Cre. Orange arrowheads highlight OTR+ neurons. Scale bar = 25µm. **C.** Overview image of the injection site highlights the presence of OTR+ astrocytes outside of the GFP injection (white arrowhead). The presence of OTR+ neurons remain unaffected by the viral injection (orange arrowheads). Scale bar = 100µm. **D.** Bar graphs show a significant reduction in OTR astrocytes, but not neurons within the injection site of OTR fl/fl mice injected with AAV_GFAP_GFP_Cre. *** p<0.001, **** p<0.0001 t-test.

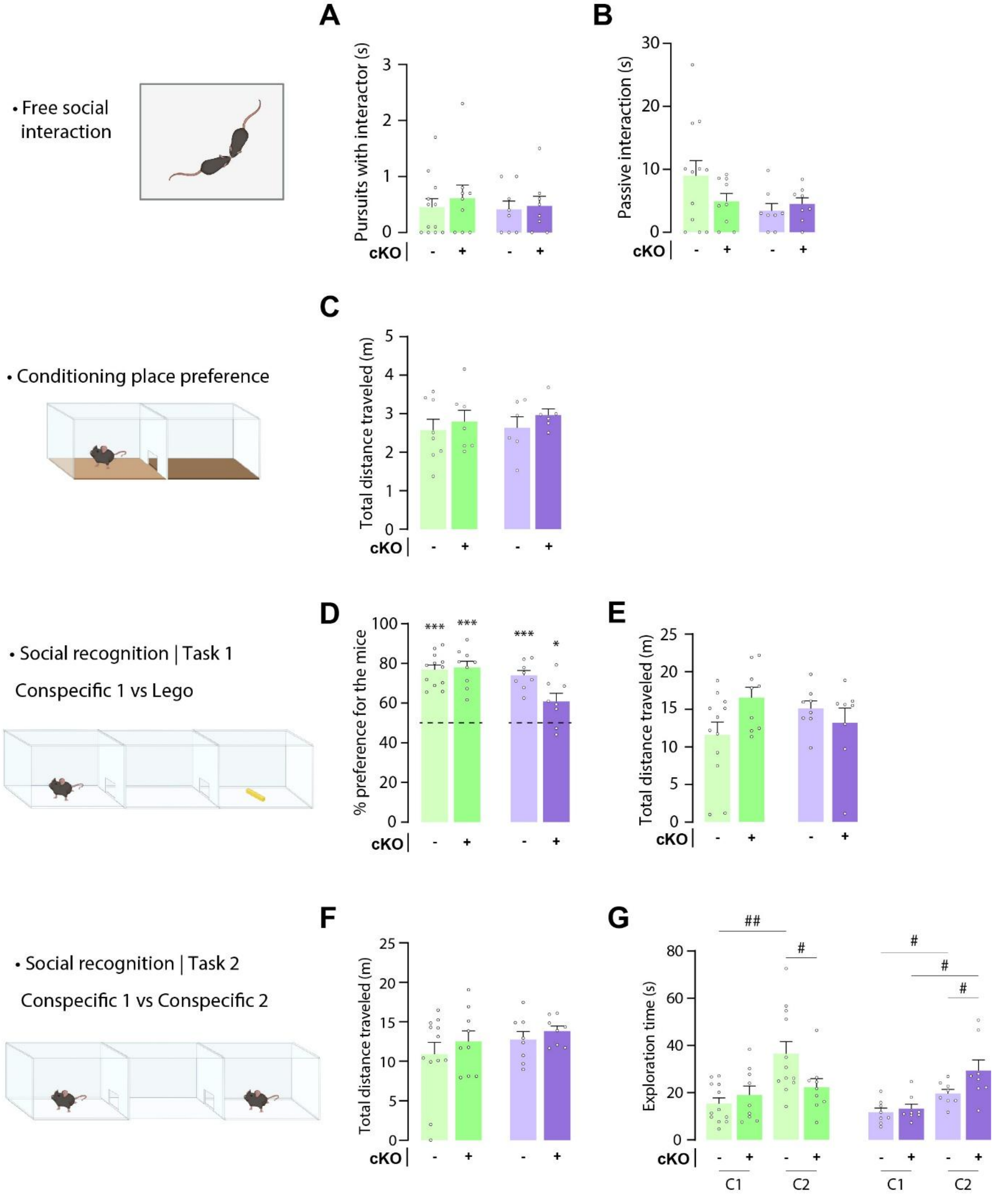

<u>Figure S8</u>: Involvement of NAc astrocytic OTR in mice social behaviors

A. Bar plot showing mean time of pursuits interaction in male and female mice (n_{CTRL} ♂= 12, $n_{OTR\ cKO}$ ♂= 9, n_{CTRL}♀= 8, $n_{OTR\ cKO}$ ♀= 8). **B**. Bar plot showing mean time of passive interaction in male and female mice (n_{CTRL} ♂= 12, $n_{OTR\ cKO}$ ♂= 9, n_{CTRL}♀= 8, $n_{OTR\ cKO}$ ♀= 8). **C.** Bar plot showing mean total distance traveled in both arms (n_{CTRL} ♂= 8, $n_{OTR\ cKO}$ ♂= 7, n_{CTRL} ♀= 6, $n_{OTR\ cKO}$ ♀= 6) **D.** Bar plot showing mean percentage of preference for the conspecific during social recognition test (n_{CTRL} ♂= 12, $n_{OTR\ cKO}$ ♂= 9, n_{CTRL} ♀= 8, $n_{OTR\ cKO}$ ♀= 8). **E**. Bar plot showing mean total distance

traveled during task 1 of social recognition test ($n_{CTRL ♂}$= 12, $n_{OTR\ cKO ♂}$= 9, $n_{CTRL ♀}$= 8, $n_{OTR\ cKO ♀}$= 8). **F**. Bar plot showing mean total distance traveled during task 2 of social recognition test ($n_{CTRL ♂}$= 12, $n_{OTR\ cKO ♂}$= 9, $n_{CTRL ♀}$= 8, $n_{OTR\ cKO ♀}$= 8). **G**. Bar plot showing mean exploring time with each conspecific (1 or 2) ($n_{CTRL ♂}$= 12, $n_{OTR\ cKO ♂}$= 9, $n_{CTRL ♀}$= 8 $n_{OTR\ cKO ♀}$= 8). Data are expressed as mean across animal ± SEM. Detailed statistics can be found in *Statistic Table 6*. # p<0.05, ## p<0.01, ### p<0,001 for SHAM vs OTKo comparison with two way Anova and sidak's or tukey's post hoc test, & * p<0.05, ** p<0.01, *** p<0,001, for one sample t-test against chance (50%).

Extended Data Table 1: List of reagents

Experiments	Antigen / Target	Company	Company reference	Final concentration	medium
	GFAP	abcam	ab53554	1:1000	
	Glutamine Synthetase	abcam	ab176562	1:500	
	S100b	Synaptic Systems	287 004	1:500	
IHC	OXTR	Alomone labs	AVR-013	1:100	
	OXTR1	Froemke lab		1:250	1x PBS
	Cre	abcam	ab190177	1:500	
	Oxytocin Receptor	Alomone	AVR-013	1:200	
	Alexa Fluor 488 anti rabbit	Invitrogen	A11008	1:1000	
	Alexa Fluor 488 anti rabbit	Invitrogen	A21206	1:1000	
	Alexa Fluor 488 anti goat	Invitrogen	A11055	1:1000	
	Alexa Fluor 488 anti guinea pig	Invitrogen	A11073	1:1000	
	Alexa Fluor 594 anti rabbit	Invitrogen	A21207	1:1000	
	Alexa Fluor 647 anti rabbit	Invitrogen	A31573	1:1000	
	Alexa Fluor 647 anti goat	Invitrogen	A21447	1:1000	
	Alexa Fluor 647 anti guinea pig	Invitrogen	A21450	1:1000	
RNAscope					
	**Name of reagent**	**Reference**			
	Mm Oxtr	412171			
	Mm GFAP-C2	313211-C2			
	3-plex Negative Control Probe	320871			
	3-plex Positive Control Probe - Mm	320881			
	Rn Oxtr	483671			
	3-plex Positive Control Probe - Rn	320981			Wash buffer
	TSA Vivid Flurophore Kit 520	7523			
	TSA Vivid Flurophore Kit 570	7526			
	TSA Vivid Flurophore Kit 650	7527			
	TSA buffer	322809			

Experiments	Virus name	Full name	Company	Company reference	Concentration	Injected volume	medium
Viruses stereotaxic injections	GFAP-GCaMP6s	AAV1.GfaABC1D-GCaMP6s	IGBMC	A238	1E+13	200 nL	
	GFAP-GFP-IRES-Cre	rAAV1/2-gfaABC1D-GFP-IRES-Cre	IGBMC	A270	1E+13	300 nL/nucleus	PBS

Buffer solution	Name	Full name	Company	Company reference	Concentration	medium
	HCl	Hydrochloric acid	Termo fischer scientific	H1200PB15	93	aCSF Bath medium
	NMDG	N-methyl-D-glucamine	Sigma-Aldrich	M2004	93	aCSF Bath medium
	KCl	Potassium chloride	Carlo erba	471177	2,5	aCSF Bath medium
	NaH2PO4	Sodium phosphate monobasic monohydrate	Sigma-Aldrich	S9638	1,25	aCSF Bath medium
	NaHCO3	Sodium bicarbonate	Sigma-Aldrich	S5761	30	aCSF Bath medium
	HEPES	4-(2-Hydroxyethyl)piperazine-1-ethanesulfonic acid, N-(2-Hydroxyethyl)piperazine-N′-(2-ethanesulfonic acid)	Sigma-Aldrich	H4034	20	aCSF Bath medium
	NaCl	Sigma-Aldrich	793566	10	25	aCSF Bath medium
					5	aCSF Bath medium

	Thiourea		Sigma-Aldrich	T8656	2	aCSF Bath medium
	Sodium Pyruvate	ketopropionic acid sodium salt	Sigma-Aldrich	P2256	3	aCSF Bath medium
	N-Acetyl-L-Cysteine		Sigma-Aldrich	A7250	10	aCSF Bath medium
	Kynurenic acid		Sigma-Aldrich	K3375	2,5	aCSF Bath medium
	MgSO4.7H2O	Magnesium sulfate hydrate	Merck	105886	10	aCSF Bath medium
	CaCl2.2H20				0,5	aCSF Bath medium

**Calcium imaging on slices**	**Name**	**Full name**	**Company**	**Company reference**	**Concentration**
	Kolliphor® EL	Polyoxyl 35 hydrogenated castor oil	Sigma-Aldrich	C5135	
	OGB1-AM	Oregon Green BAPTA 1 acetoxymethylesther	Thermo Fisher Scientific	O6807	20µM
	Pluronic F127	/	Sigma-Aldrich	P2443	
	Sulforhodamine 101	/	Sigma-Aldrich Sigma-Aldrich	S7635	1 µM in ACSF
	TGOT	(Thr^4,Gly^7)-Oxytocin ; H-Cys-Tyr-Ile-Thr-Asn-Cys-Gly-Leu-Gly-NH_2	Bachem Abcam	H-7710	0,5 µM in ACSF
	TTX citrate	Octahydro-12-(hydroxymethyl)-2-imino-5,9:7,10a-dimethano-10aH-[1,3]dioxocino[6,5-d] pyrimidine-4,7,10,11,12-pentol + citrate buffer	Bachem Abcam	ab120055	1 µM in ACSF
	dOVT	d(CH2)51,Tyr(Me)2,Thr4,Orn8,des-Gly-NH29)-Vasotocin	Bachem Abcam		1 µM in ACSF
	SR49059	/	Bachem Abcam		10 nm in ACSF

**Drugs**	**Name**	**Commercial name**	**Concentration**	**Administration**
	Ketamine	Imalgene	100 mg/kg	IP
	Xylazine	Rompun	20 mg/kg	IP
	Lidocaine	Lurocaine	10 mg/kg (before perfusion) or 2 mg/kg (during surgery)	SC
	Bupivacaine	/	2 mg/kg	SC
	Metacam	/	10 mg/kg	SC
